# A highly sensitive carbon quantum dots sensor for hypochlorite ion detection through fluorescence-off method

**DOI:** 10.1039/d6ra05419f

**Published:** 2026-10-14

**Authors:** Ankita Dutta, Sahidul Islam, Ujjwal Mandal

**Affiliations:** a Department of Chemistry, The University of Burdwan Golapbag Burdwan-713104 India; b Department of Chemistry, Murshidabad Maharaja Krishnanath University Berhampore 742101 India umandal@chem.buruniv.ac.in

## Abstract

Hypochlorite ions have a key role in the biological immune system. As soon as the body detects an attack by bacteria or viruses, it produces, by some complex reaction mechanism, hypochlorite ions (ClO^−^). These ions are very powerful oxidizing agents and can kill bacteria or viruses by digesting their (virus, bacteria) constituent proteins or other biomolecules. Due to this, detection of hypochlorite ions is very important. Here we have synthesized Cu-doped CQDs from succinic acid and cupric acetate through a simple aqueous reflux method at 90 °C. The prepared CQD showed good fluorescence properties (quantum yield (QY) ∼31.33%). Hypochlorite ions quenched the emission intensity of the CQD, exclusively. The probe was good enough to detect a very low concentration of the hypochlorite ions, as low as 5.76 nM. Synthesized CQDs can be used to sense hypochlorite ions in real water samples also. The linear range of the detection was 0–400 nM. The prepared CQDs have been characterized by UV-vis spectrophotometer, fluorimeter, FT-IR (Fourier transform infrared spectroscopy), Dynamic Light Scattering, XRD (X-ray Diffraction) and TEM (transmission electron microscopy), EDX, and TCSPC. From the TEM images it is clear that the CQDs are spherical. We measured their diameters to be in the range 2–5 nm.

## Abstract 2

Hypochlorite ions have a key role in the biological immune system.

## Graphical abstract

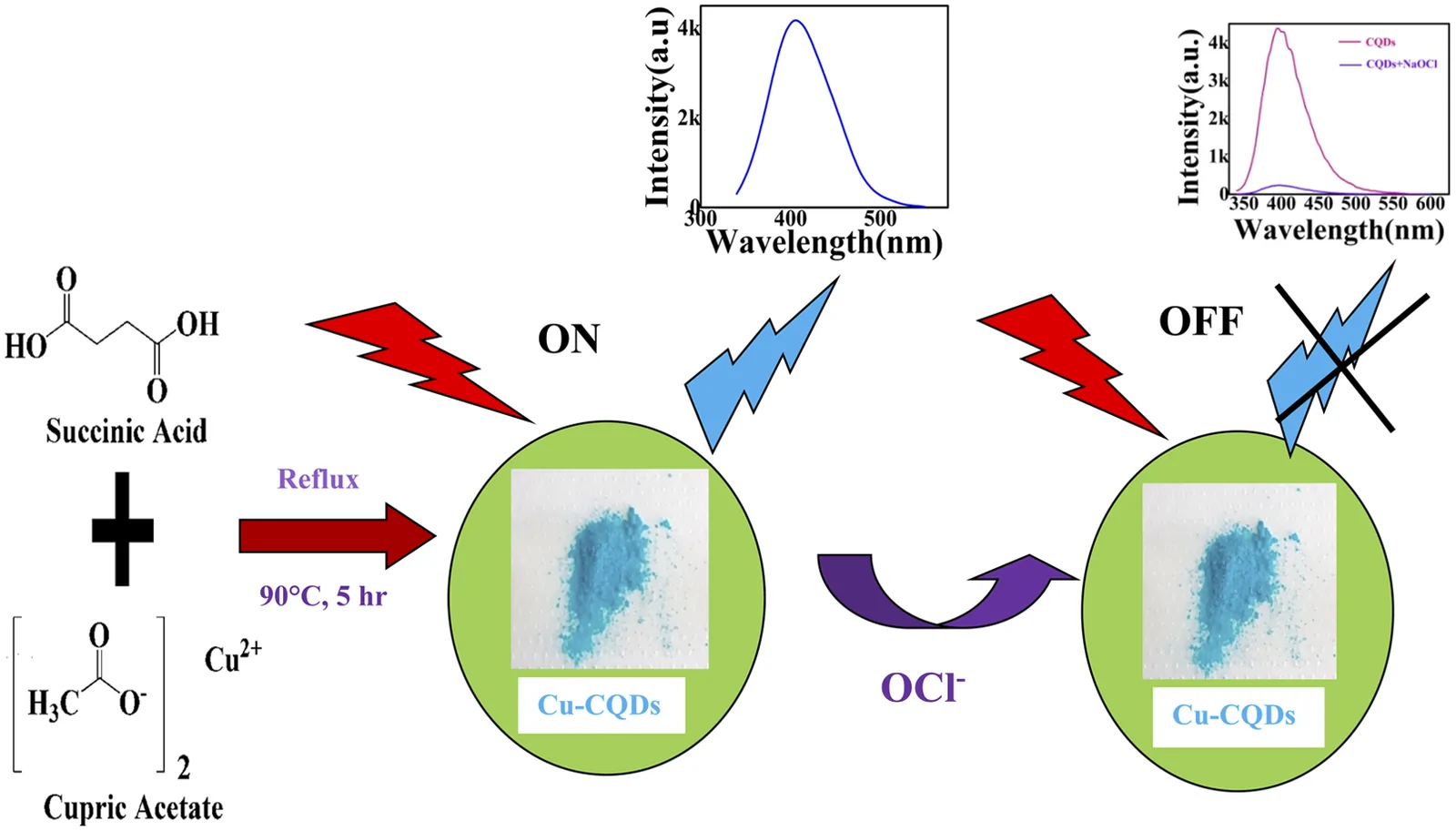

## 1. Introduction

Quantum dots (QDs) are zero-dimensional semiconductor nanomaterials that exhibit unique size-dependent optical and electronic properties arising from quantum confinement.^1^ Their fluorescence properties have led to extensive applications in bioimaging,^2,3^ sensing,^4^ pathogen detection^5^ and optoelectronics.^6^ However, conventional QDs may contain toxic heavy metals such as Cd and Pb, which can restrict their use in biological and environmental applications.^7^ Carbon quantum dots (CQDs), owing to their comparatively low toxicity,^8^ good water solubility,^9^ photostability,^10^ biocompatibility^9^ and tunable photoluminescence properties,^11^ have therefore emerged as attractive alternatives for fluorescence-based sensing applications.^12,13^

Among the various applications of CQDs, fluorescence sensing of reactive chemical species is particularly attractive because fluorescence measurements offer high sensitivity, operational simplicity, and rapid response. Hypochlorite (OCl^−^) is an important reactive oxygen species^14^ involved in the biological immune response,^15^ where it participates in the destruction of invading microorganisms.^16,17^ At the same time, hypochlorite is extensively used as a disinfectant and bleaching agent^18^ However, excessive levels of OCl^−^ can cause oxidative damage and have been associated with adverse biological effects.^19^ Therefore, the development of sensitive and selective methods for monitoring OCl^−^ is important for both biological and environmental applications.

Fluorescence-based detection is particularly suitable for OCl^−^ because changes in fluorescence intensity can be monitored conveniently at very low analyte concentrations.^20^ Both small-molecule fluorescent probes and quantum-dot-based probes have been reported for OCl^−^ detection.^21,22^ However, the synthesis of many small-molecule probes involves relatively complicated synthetic procedures.^23^ In contrast, CQDs can be prepared through comparatively simple and cost-effective routes and can be readily dispersed in aqueous media.^24^ These characteristics make CQDs attractive platforms for developing fluorescence-based OCl^−^ sensors.^25^ The sensing performance of CQDs can be further modified through heteroatom or metal-ion doping.^26^ Incorporation of suitable dopants can alter the electronic structure, surface states and photoluminescence properties of CQDs and thereby influence their sensing behaviour.^27^ Transition-metal ions are of particular interest because their electronic structures can provide additional surface-active sites and modify the energy states of CQDs.^28^ Copper was selected in the present study because Cu is an important biologically relevant element and copper-containing compounds have been used in a variety of chemical applications.^29^ On this basis, we considered that incorporation of Cu into CQDs could provide a suitable fluorescent platform for interaction with an oxidizing analyte such as OCl^−^. Succinic acid was selected as the carbon precursor and Cu(OAc)_2_·H_2_O as the copper source for the preparation of Cu-doped CQDs. The choice of these precursors enables the preparation of the fluorescent Cu-CQD material through a simple aqueous reflux process. Importantly, the resulting Cu-CQDs provide a fluorescent surface whose response toward OCl^−^ can be investigated through changes in fluorescence intensity.

Therefore, the objective of this work was to develop a simple, sensitive and selective fluorescence-based sensor for the detection of OCl^−^ using Cu-doped carbon quantum dots prepared from succinic acid and Cu(OAc)_2_·H_2_O. The synthesized Cu-CQDs were systematically characterized, and their fluorescence response toward OCl^−^ was investigated under optimized conditions. The mechanism responsible for fluorescence quenching was further examined using steady-state absorption/fluorescence measurements and time-resolved fluorescence studies. Finally, the applicability of the proposed Cu-CQD probe for OCl^−^ determination was evaluated using real water samples.

## 2. Characterizations

The absorption spectra of CQDs in aqueous medium were recorded using a UV-vis 2450 spectrophotometer (Shimadzu, Japan). Quartz cuvettes of path length 1 cm are used for both absorption and emission studies as a sample holder. Distinct absorption maximum was found for CQDs. The emission and excitation spectra were measured through Hitachi F-7000 spectrofluorimeter. The fluorescence quantum yield (QY) of CQDs was measured using quinine sulphate as a reference. Transmission Electron Microscope (TEM) is used to determine size and shape of CQDs which has 120 kV accelerating voltage. The TEM specimen was prepared in a given volume of CQDs dispersion. The specimen solution was dropped on a carbon coated copper grid, and to dry the sample copper grid was kept in a vacuum oven for overnight. Dynamic light scattering (DLS) [Malvern Zetasizer Nano ZS with 633 nm CW laser] was used to detect the size of the Cu-CQDs. X-ray Diffraction (XRD) spectra were studied by using a Brucker D8 Advance Diffract meter with Ni-filtered Cu Kα radiation. Fourier transform infrared (FT-IR) spectra were recorded by Shimadzu FTIR [model IR Prestige 21 CE] in the range of 400 to 4000 cm^−1^ in KBr pellets. To understand the quenching mechanism of CQDs in presence ofOCl^−^ we measured the excited state lifetime of CQDs in absence and presence ofOCl^−^. Time-Correlated Single Photon Counting (TCSPC) setup equipped with the Deltaflex Modulator Fluorescent lifetime system manufactured by Horiba Jobin Yvon was used for time-resolved fluorescence measurements. A 373 nm pulse diode was used as an excitation source. A low concentration of Ludox, a colloidal silica, was used to assess the Instrument Response Function (IRF). A full width at half maximum (FWHM) of 160 ps were obtained. Decay transients were analyzed using the supplied program EzTime (Horiba Scientific v3.2.9.6). Emission polarizer was kept at magic angle (54.7°), with respect to the excitation polarizer and a photomultiplier tube (PMT) was used for photon detection.

Following fitting function was used:1
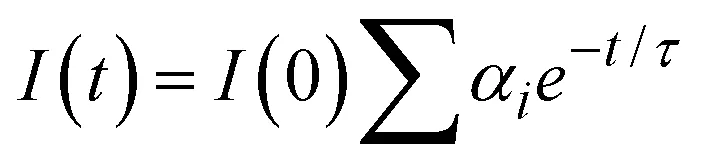


*I*(*t*) and *I*(0) are the fluorescent intensity at time *t* and 0 respectively, *t* is the time, *α* and *τ* are the contributing amplitude and its corresponding lifetime. The reduced chi-squared value was used to assess whether a fit is satisfactory or not.

## 3. Experimental section

### 3.1. Chemicals and reagents

Succinic Acid (S.A), Cupric acetate (Cu(OAc)_2_·H_2_O) was purchased from Merck, India. These chemicals were used without any further purification. Throughout the experiment whenever required we used DI water. The salts of various ions like (Ag^+^, K^+^, Ca^2+^, Cu^2+^, Ni^2+^, Ba^2+^, Pb^2+^, Hg^2+^, Cd^2+^, Co^2+^, Fe^2+^, Zn^2+^, Fe^3+^ and anions like OCl^−^, COO^−^, BO_3_^3−^, AsO_3_^3−^, Cl^−^, Br^−^, F^−^, PO_4_^3−^, N^3−^, HPO_4_^2−^, CrO_4_^2−^, SO_4_^2−^, AsO_4_^3−^, CO_3_^2−^, HSO_4_^−^, OAc^−^, SCN^−^, C_2_O_4_^2−^, NO_3_^−^, NO_2_^−^, HCO_3_^−^, SO_3_^2−^, ClO_4_^−^, AsO_2_^−^, MnO_4_^−^, Urea, CN^−^, H_2_O_2_, O_2_^−^), were purchased from Merck, India.

### 3.2. Synthesis of Cu-CQDs

In this experiment, 0.3522 g Succinic acid (SA) was dissolved in 38 mL of water, and then 2 mL of 0.1 M Cu(OAc)_2_·H_2_O solution was added. The mixture was stirred at room temperature for 10 min, and then refluxed at ∼90 °C for 5 h. The resulting product was cooled to room temperature and purified by centrifugation at 6000 rpm for 15 min to retain the supernatant. We obtained an indigo blue colored supernatant, which was filtered through 0.22 µM membrane. This as-prepared Cu-CQD solution was solidified through rotavapour at 75 °C and got indigo blue colour solid Cu-CQDs. The isolated Cu-CQD product was weighed to determine the experimental product yield. Since carbonization of succinic acid does not necessarily result in quantitative conversion of all precursor carbon into CQDs, the yield was determined from the mass of the recovered purified Cu-CQD material rather than by assuming complete carbon conversion. The isolated product yield was calculated using.

Isolated product yield (%) = (mass of recovered purified Cu-CQDs/mass of precursor used) × 100.

Isolated product yield (%) = (0.047 g/0.3922 g) × 100 = 12%.

Under the present synthetic conditions, the isolated yield of the purified Cu-CQDs was found to be 12% (Scheme 1).

**Scheme 1:**
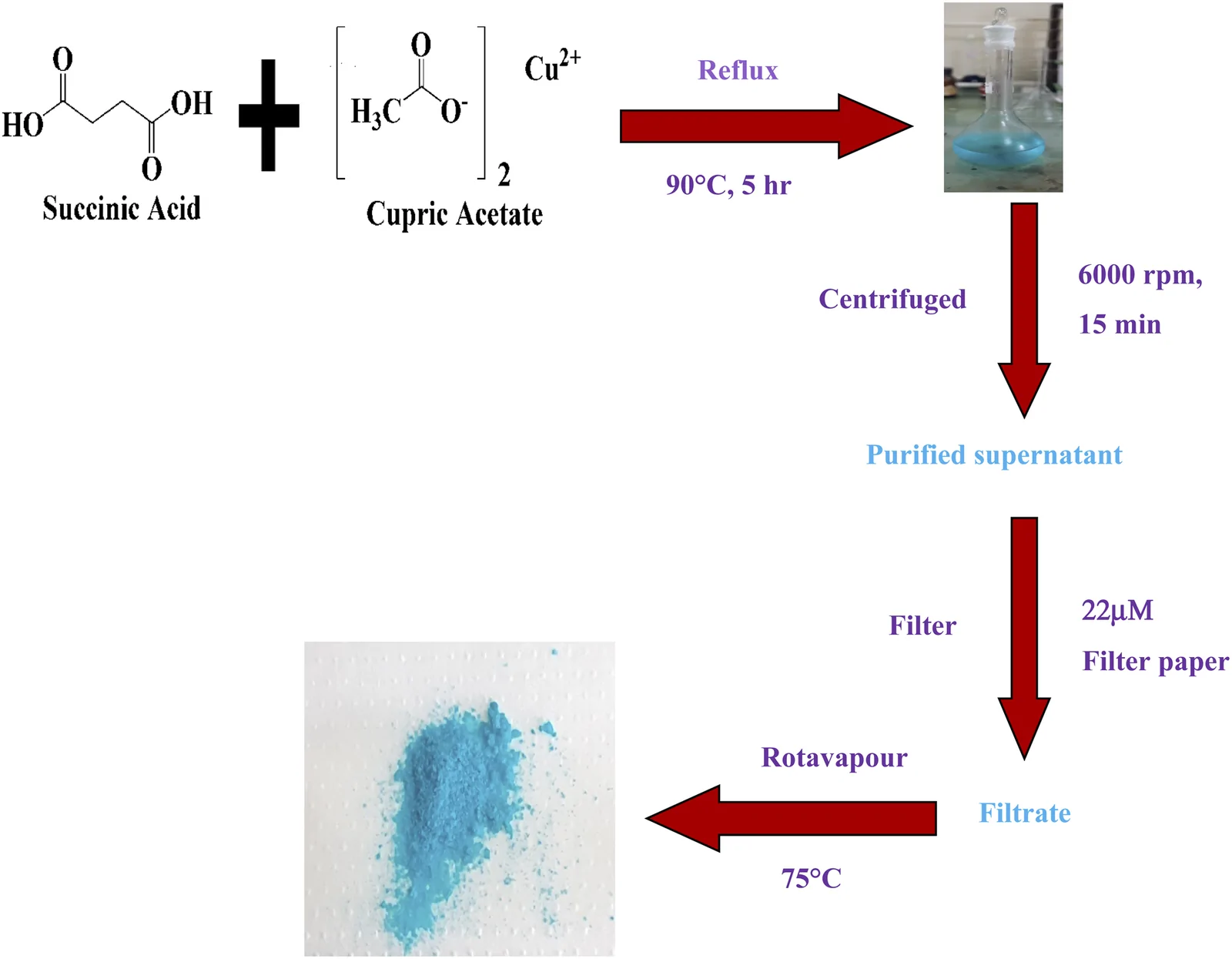
Synthesis of copper doped carbon quantum dots.

### 3.3. Quantum yield calculation

Carbon quantum dots prepared from succinic acid and cupric acetate works as a turn off fluorescence sensor. The fluorescence intensity decreases rapidly on addition of sodium hypochlorite. Here, we have utilized quinine sulphate (reported QY 0.54) as a reference to calculate the fluorescence quantum yield (QY) of Cu-CQDs. To measure the quantum yield of Cu-CQDs, the following equation^30^ is used:2
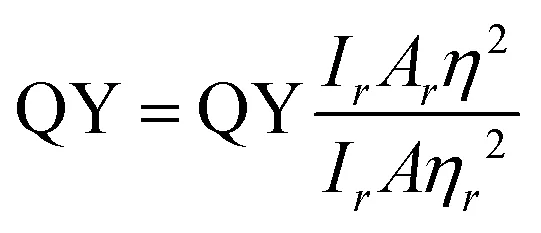
Here, the QY_r_ and QY represented the quantum yield of the reference and the quantum yield of the sample, respectively. The area integrated emission spectrum of the reference is symbolized as *I*_r_ and that of the sample is symbolized as *I*. Absorbance of reference and sample are represented as *A*_r_ and *A*, respectively. *η*_r_ and *η* are the viscosity coefficients of the medium of reference and the sample (Table 1).

**Table 1:** Quantum yields of Cu-CQDs in water

Probe	Medium	Absorbance	Emission/area integrated	Quantum yield
C 480	Water	0.11	409 672.98	0.54
Cu-CQDs	Water	0.14	302 602	0.3133

### 3.4. pH effect on fluorescence emission of Cu-CQDs

We have recorded the pH dependence of the Cu-CQDs' emission intensity. Fig. 1 shows the maximum emission intensity of the Cu-CQDs solution at pH 8. Hence, we performed the entire experiment at pH 8 using sodium phosphate buffer.

**Fig. 1:**
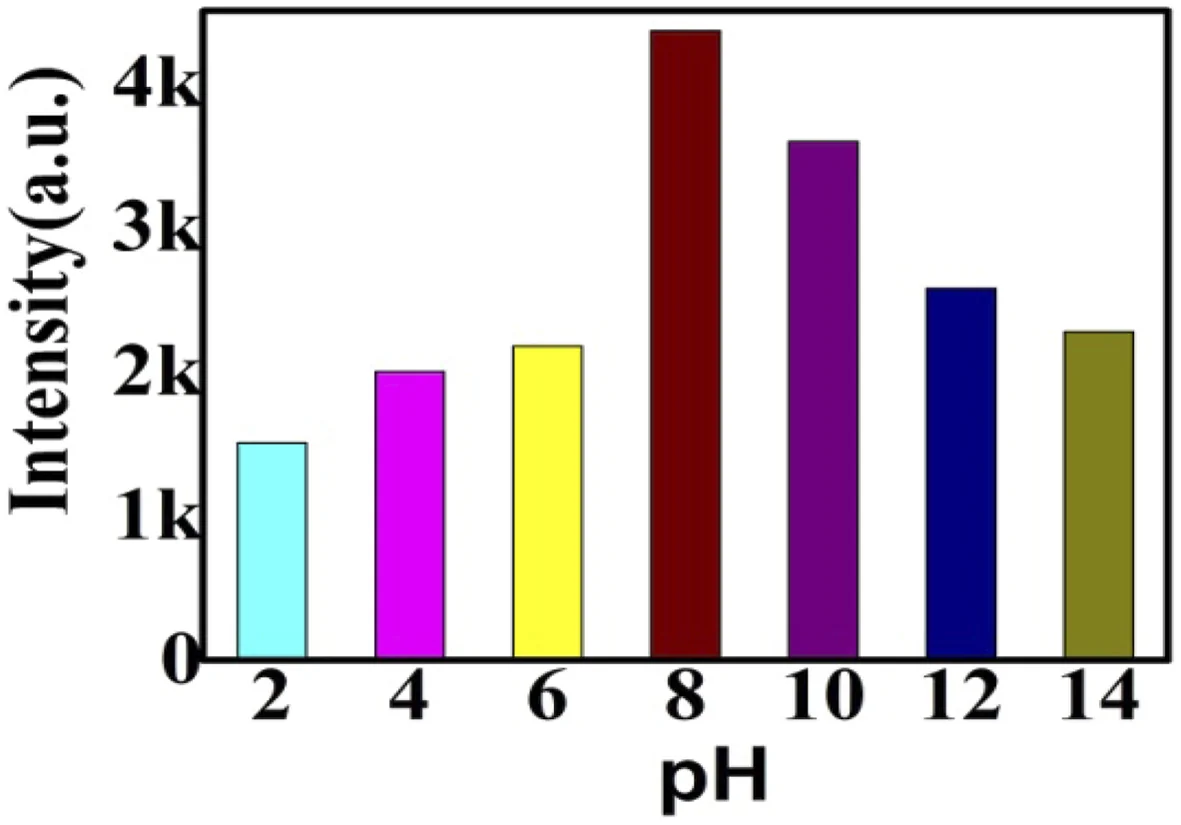
Emission intensity of Cu-CQDs at different pH values.

### 3.5. Detection of OCl^−^

In this work the Cu-CQDs are used to detect ions. The fluorescence data of CQDs in the presence of various metal ions like Ag^+^, K^+^, Na^+^, Mg^2+^, Mn^2+^, Ca^2+^, Cu^2+^, Ni^2+^, Ba^2+^, Pb^2+^, Hg^2+^, Cd^2+^, Co^2+^, Fe^2+^, Zn^2+,^Fe^3+^, and anions like OCl^−^,COO^−^, BO_3_^3−^, AsO_3_^3−^, Cl^−^, Br^−^, F^−^, PO_4_^3−^, N_3_^−^, HPO_4_^2−^, CrO_4_^2−^, SO_4_^2−^, AsO_4_^3−^, CO_3_^2−^, HSO_4_^−^, OAc^−^, SCN^−^, C_2_O_4_^2−^ , NO_3_^−^, NO_2_^−^, HCO_3_^−^, SO_3_^2−^,ClO_4_^−^, AsO_2_^−^, MnO_4_^−^, Urea, CN^−^, H_2_O_2_, O_2_^−^, under the same condition were studied.

### 3.6. Detection of OCl^−^ in a real water sample

We have collected tap water from our laboratory and lake water from the lahar of Burdwan University as a real sample. The tap water and lake water were spiked with different concentrations of OCl^−^and Cu-CQDs.^31^ The fluorescence intensity of Cu-CQDs was recorded with the fluorescence spectrophotometer. The recoveries were calculated according to eqn (1)3
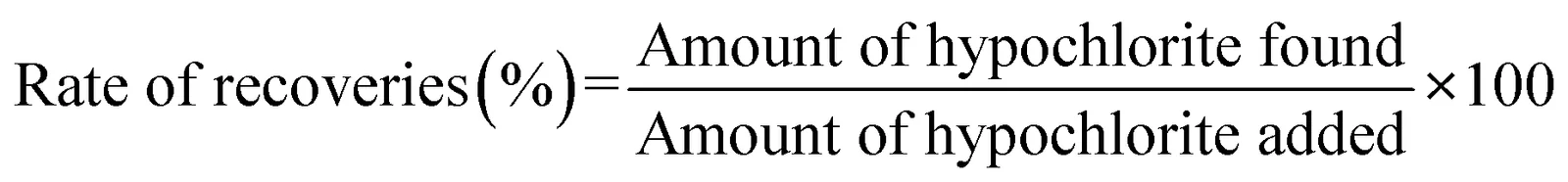


## 4. Results

### 4.1. TEM and DLS study

TEM is used to determine the shape and size of synthesized Cu doped CQDs. From Fig. 2 (inset), very clear spherical shapes of CQDs are seen. The diameter of the prepared CQDs varies from 2 to 5 nm. From the size distribution histogram of Cu-CQDs (Fig. 2), we can see that the maximum no of particles has a size of 3 nm. To confirm the size of synthesized Cu doped CQDs, we have studied dynamic light scattering (DLS), and Fig. 3 shows that the size of Cu-CQDs lies between 3.6 nm and 4.17 nm, which correlates with the TEM data. This result indicates that the Cu-CQDs do not agglomerate.

**Fig. 2:**
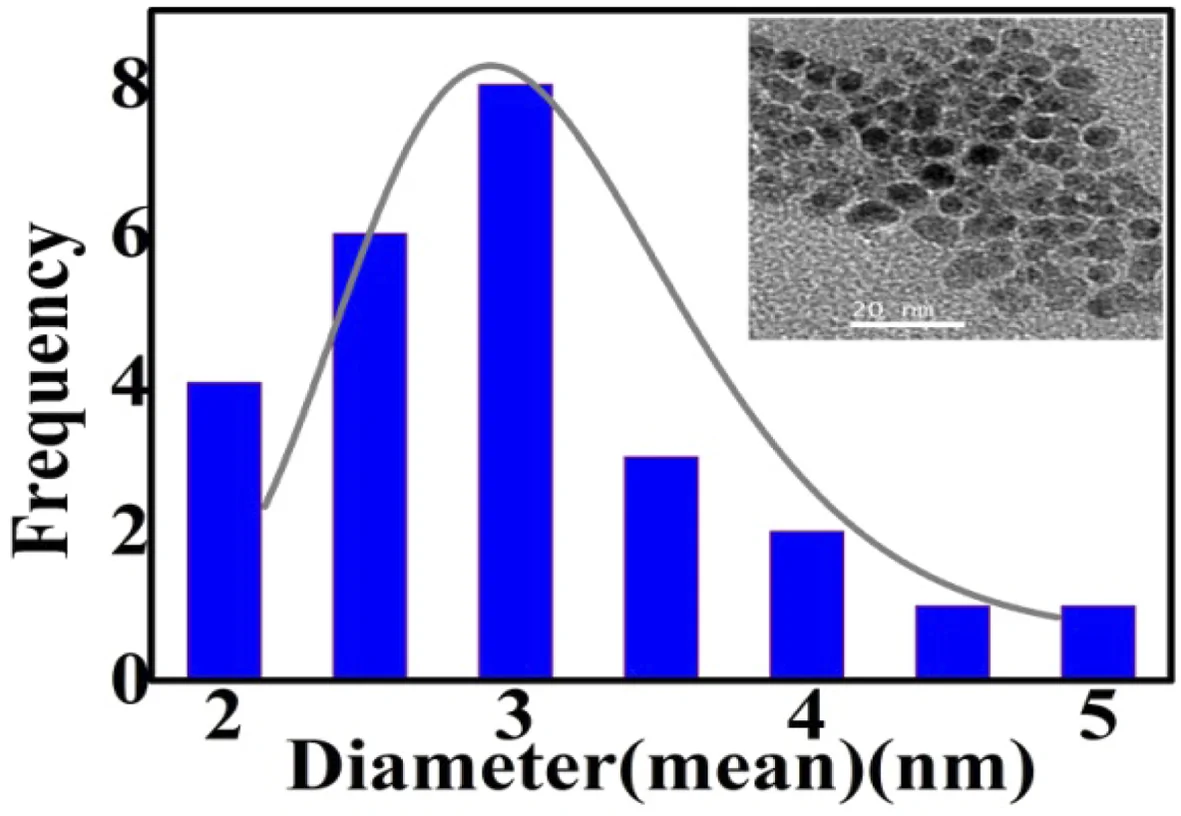
TEM image and size distribution histogram of synthesized Cu-CQDs.

**Fig. 3:**
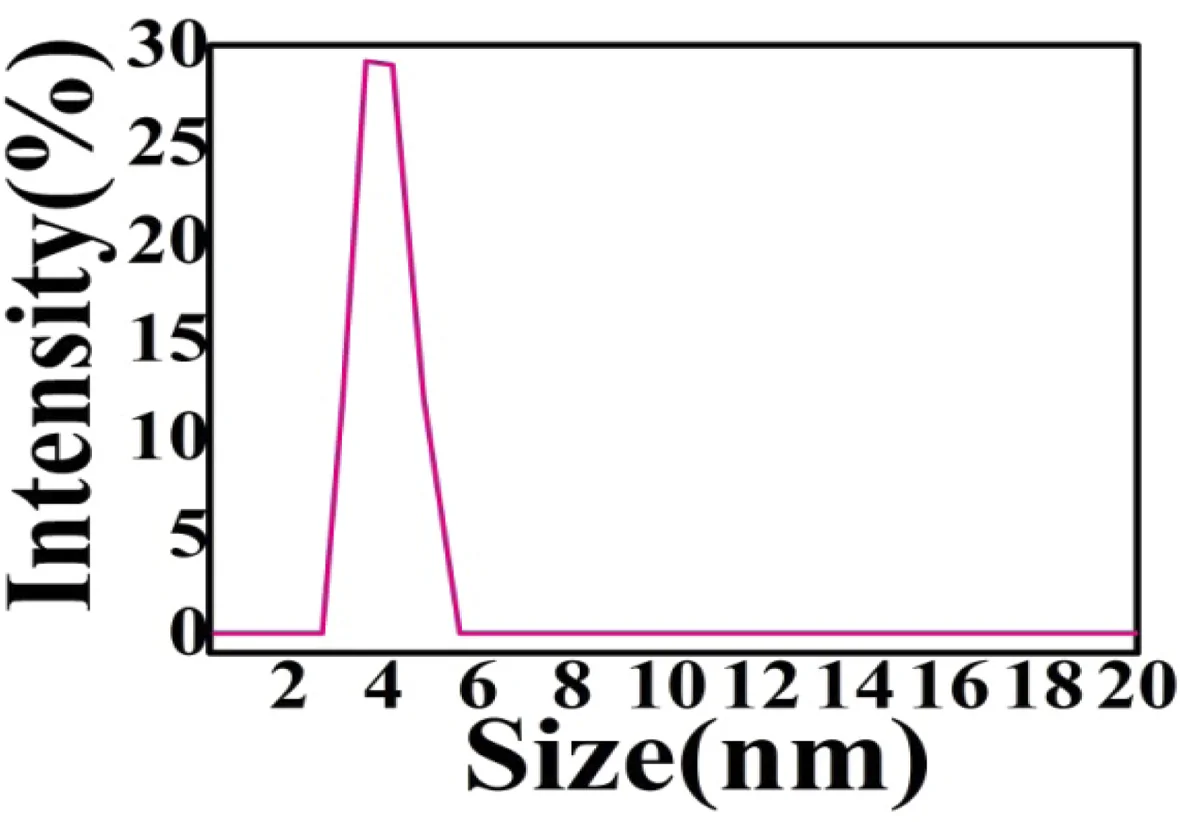
Dynamic light scattering of Cu-CQDs.

### 4.2. X-ray diffraction study

Fig. 4 shows the XRD pattern of synthesized Cu doped CQDs. The XRD characteristics peaks appear at (2*θ*) = 11.7°, 20.1°, 26.2°, 31.4°, 43.4°and 50.6°. The two peaks noticed at 43.4° and 50.6° are assigned to (111) and (200) planes of Cu.^32^ In the Cu-CQDs, four peaks observed at 2*θ* = 11.4°, 20.6°, 26.2° and 31.4° are characteristics of carbon atom.^33,34^

**Fig. 4:**
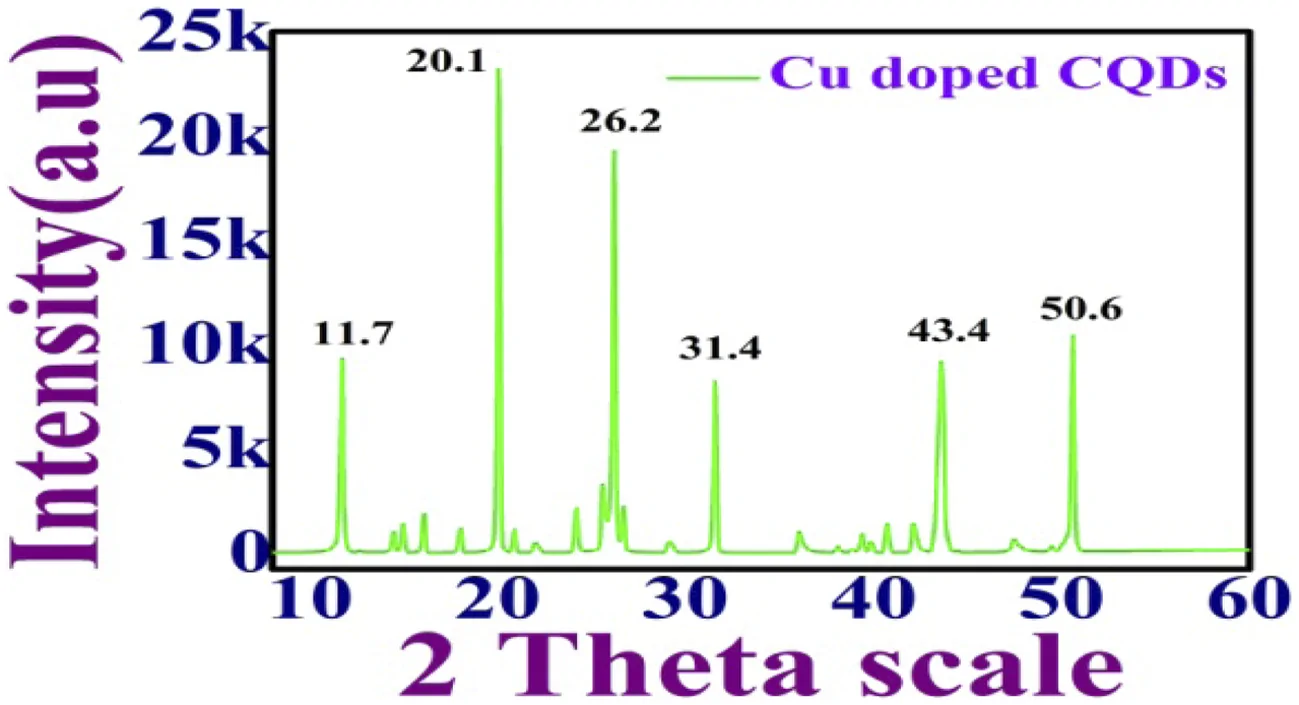
XRD pattern of Cu-CQDs.

### 4.3. EDX analysis

The composition and spatial arrangement of synthesized carbon quantum dots have been studied through EDX analysis and elemental mapping. Fig. S1 and S2 show EDX and elemental mapping, respectively. From Table 2, we can conclude that our synthesized CQDs contain Carbon, Oxygen, and Copper as element having weight percentage 49.98, 45.53, and 4.50 and atomic percentage 58.80, 40.20, and 1.00, respectively.

**Table 2:** Element percentage in Cu-CQDs obtained from EDX study

Element	Weight %	Atomic %	Net int.
C K	49.98	58.80	531.82
O K	45.53	40.20	799.12
Cu K	4.50	1.00	129.15

Elemental mapping showed the spatial distribution of these elements within the analyzed particles. These results provide compositional evidence for the incorporation of Cu into the synthesized CQDs (Table 2, Fig. S1 and S2).

### 4.4. FT-IR study

FT-IR spectroscopy was used to investigate the surface functional groups of the synthesized Cu-CQDs (Fig. 5). The broad band around 3400 cm^−1^ is attributed mainly to O–H stretching vibrations of surface hydroxyl group,^35^ while the feature around 2960 cm^−1^ may arise from stretching vibrations associated with aliphatic C–H and/or strongly hydrogen-bonded carboxylic functionalities.^36^ The bands observed around 1426 and 914 cm^−1^ are associated with vibrations of oxygen-containing^37^ and C–C^38^ containing surface groups, whereas the bands around 1179 ^39^ and 1315 cm^−1^ indicate C–O-containing functionalities.^40^

**Fig. 5:**
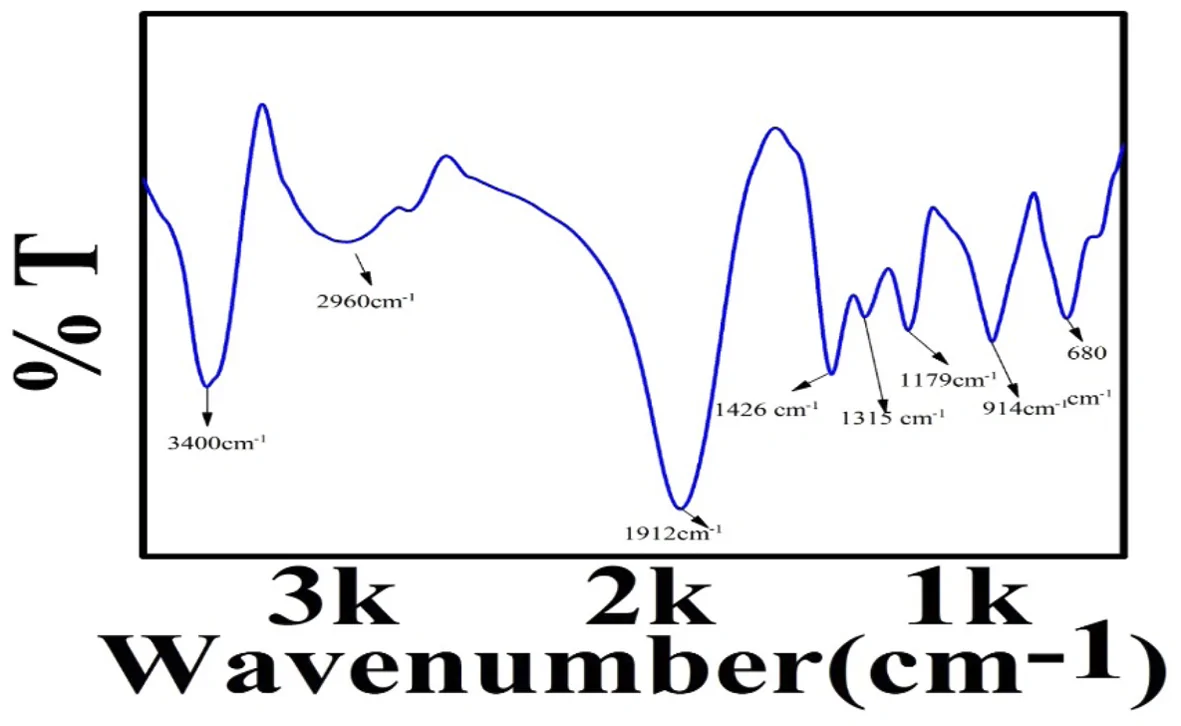
FT-IR spectra of Cu-CQDs.

A relatively sharp feature is also observed near 1912 cm^−1^. Although a band in this region may potentially arise from cumulative double-bond-containing functionalities, assignment of this peak specifically to an allene-type C=C=C group cannot be made conclusively from FT-IR spectroscopy alone.^41^ Therefore, the band is interpreted cautiously and is not used as direct evidence for graphitic-carbon formation.

The FT-IR spectrum thus indicates the presence of several oxygen-containing surface functionalities on the Cu-CQDs. The formation of the Cu-CQD material is established from the combined FT-IR, XRD, TEM, EDX and elemental-mapping results rather than from any single vibrational band or from the colour of the obtained material.

### 4.5. Absorbance

From Fig. 6, the UV-vis spectra of the Cu-CQDs show that the absorption peaks of CQDs are centered at about 246 nm. This high-energy absorption can be associated mainly with π → π* transitions involving sp^2^-conjugated carbon domains of the CQDs. In addition, oxygen-containing surface functionalities and defect-related states may introduce lower-energy *n* → π* transitions and surface-state absorption extending toward longer wavelengths.^42^ Cu incorporation can further modify the electronic and surface-state distribution of the CQDs and thereby influence their optical response.

**Fig. 6:**
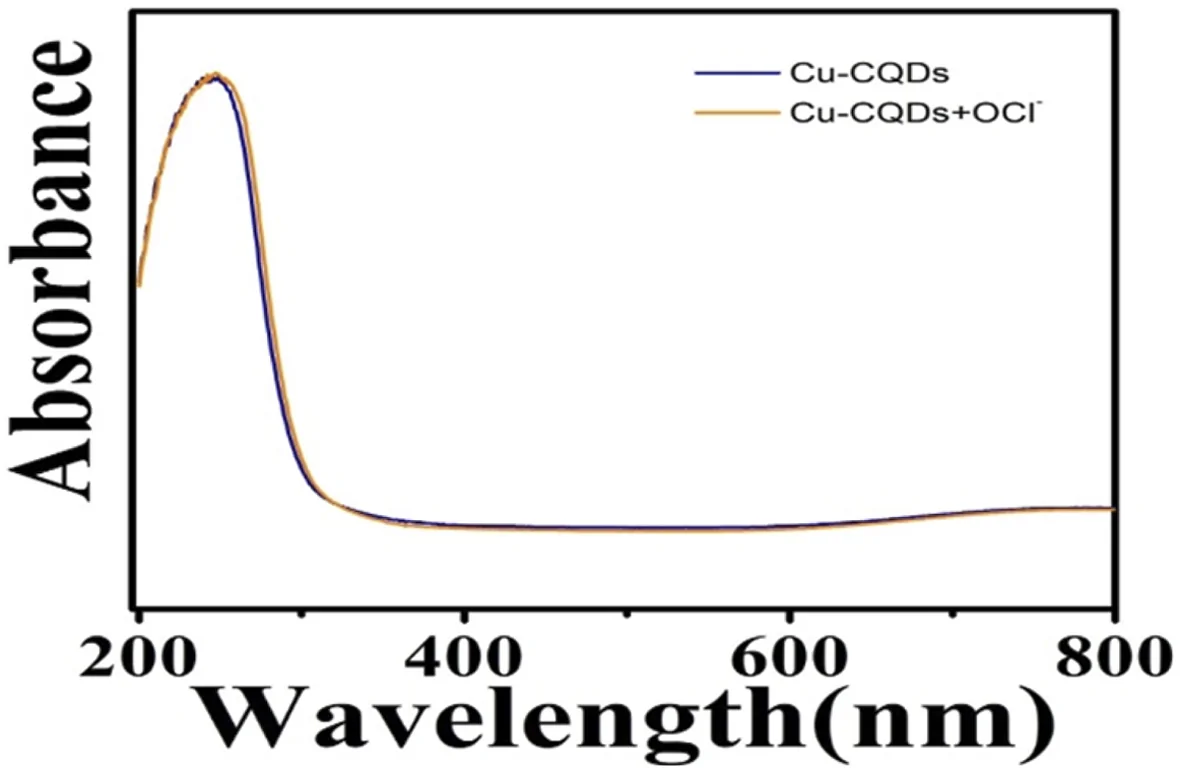
UV-vis spectra of Cu-CQDs.

The absorption spectra of Cu-CQDs were also recorded in the absence and presence of OCl^−^ (Fig. 6). No substantial new absorption band or pronounced spectral shift was observed after addition of OCl^−^, indicating the absence of a strongly absorbing new ground-state complex under the experimental conditions. The absorption results were therefore considered alongside the steady-state and time-resolved fluorescence measurements when discussing the fluorescence-quenching mechanism. From the Tauc plot the estimated band gap (Fig. 7), which is 3.40 eV, characterizes the excitation energetics of the Cu-CQDs.

**Fig. 7:**
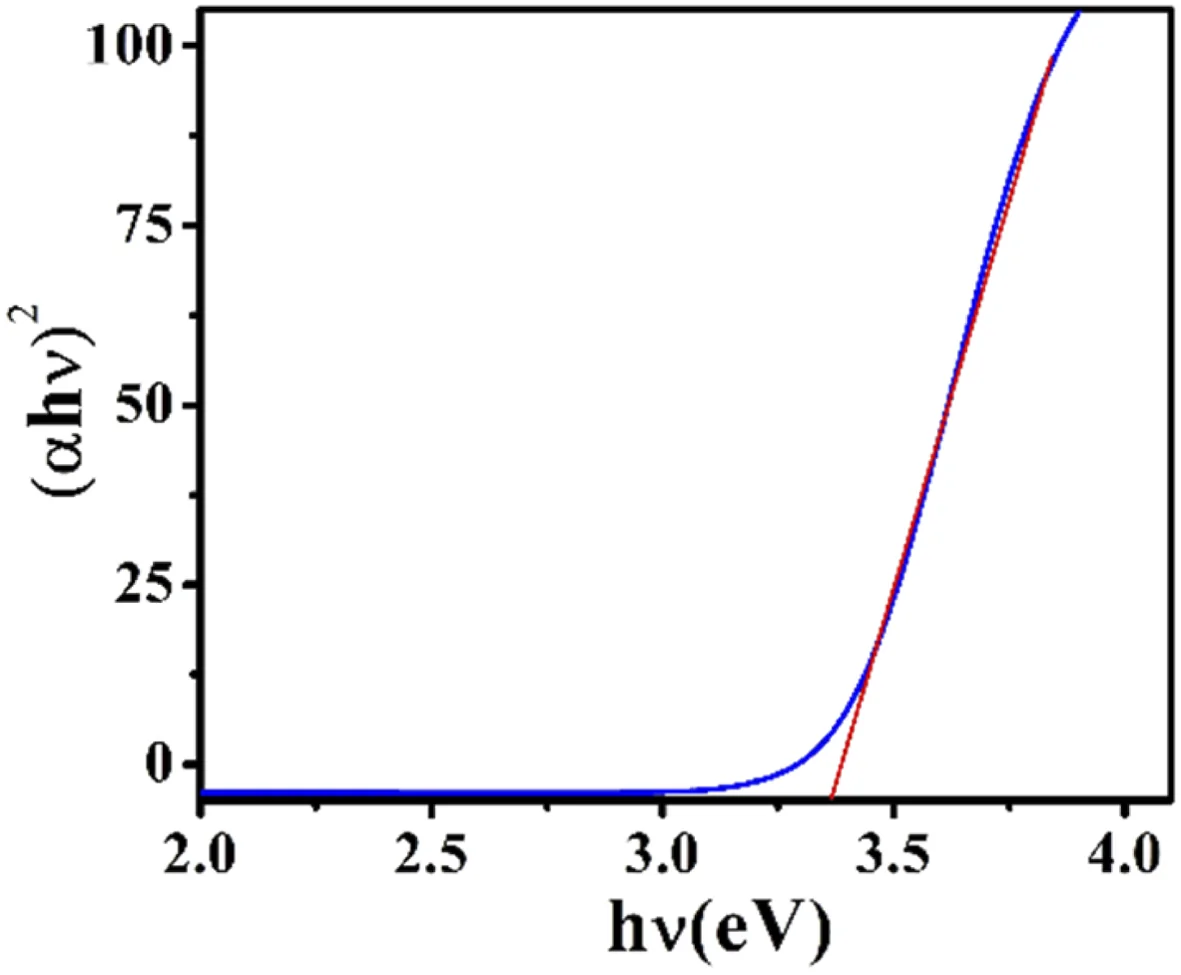
Tauc plot obtained from UV-Vis absorption spectra of Cu-CQDs.

### 4.6. Fluorescence emission study

The Cu-CQDs exhibit a broad fluorescence emission band with maximum emission around 400 nm (Fig. 8). The fluorescence intensity depends on the excitation wavelength, with the highest emission intensity observed upon excitation at approximately 340 nm. Accordingly, 340 nm was selected as the excitation wavelength for subsequent fluorescence measurements.

**Fig. 8:**
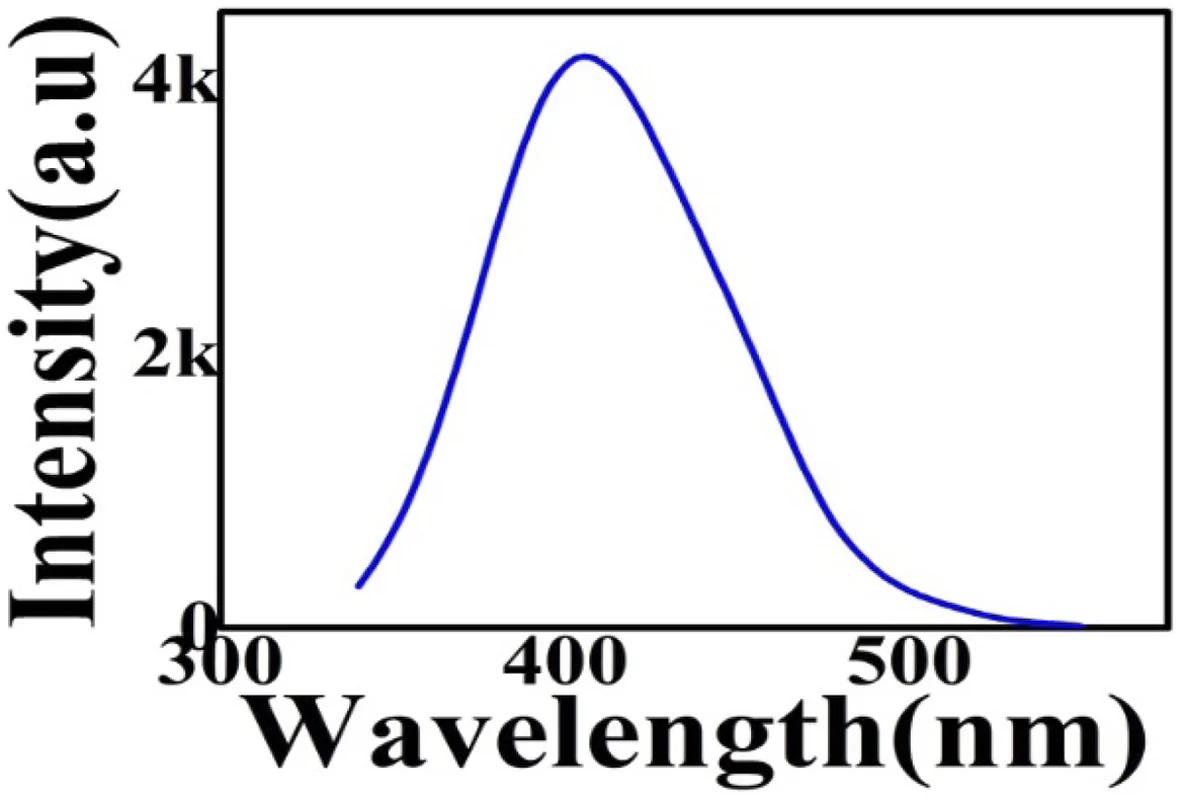
Emission spectra of Cu-CQDs.

The photoluminescence of CQDs generally originates from a combination of carbon-core electronic states and surface/defect-related emissive states rather than from a single well-defined intrinsic band-gap transition. In the present Cu-CQDs, oxygen-containing surface functionalities, sp^2^-conjugated carbon domains and Cu-associated electronic states may generate trapping and emissive states within the electronic structure of the nanoparticles. Following photoexcitation, relaxation and radiative recombination through these surface- and defect-related states can therefore contribute to the observed fluorescence emission.

Copper incorporation may modify the distribution of these surface states and associated charge-transfer pathways, thereby influencing the photoluminescence behavior of the CQDs. Such Cu-associated and surface electronic states are also relevant to the fluorescence response toward OCl^−^, since interaction with the strongly oxidizing OCl^−^ species can introduce additional non-radiative deactivation pathways for the excited Cu-CQDs. The detailed quenching mechanism is discussed in Section 5.

## 5. Detection mechanism

The fluorescence intensity of the Cu/-CQDs decreased markedly upon addition of OCl^−^, demonstrating a strong fluorescence-quenching response toward hypochlorite. To clarify the origin of this quenching, possible contributions from the inner-filter effect, static quenching and dynamic excited-state deactivation were considered.

The absorption maximum of OCl^−^ occurs at approximately 292 nm and does not significantly overlap with the principal excitation and emission regions of the Cu-CQDs under the experimental conditions. Therefore, a substantial inner-filter contribution to the observed fluorescence decrease is unlikely. In addition, the UV-visible absorption spectrum of the Cu-CQDs did not show a pronounced new absorption band or substantial spectral shift after addition of OCl^−^, providing no clear evidence for formation of a strongly absorbing ground-state complex. The corresponding control spectra and analyses are provided in the (SI).

In contrast, the time-resolved fluorescence measurements showed a clear decrease in the average fluorescence lifetime of the Cu-CQDs after addition of OCl^−^. This lifetime shortening demonstrates the introduction of an additional excited-state non-radiative deactivation pathway and supports a predominantly dynamic fluorescence-quenching process.

Considering the strong oxidizing character of OCl^−^, the fluorescence response is consistent with interaction of OCl^−^ with oxidizable surface functionalities and Cu-associated electronic states of the Cu-CQDs. Such interaction can perturb the emissive surface states and facilitate electron-transfer-assisted non-radiative deactivation of the excited Cu-CQDs. The combined steady-state and time-resolved fluorescence results are therefore consistent with a photoinduced electron-transfer (PET)-associated quenching mechanism. A schematic representation of the proposed sensing mechanism is shown in Fig. 9.

**Fig. 9:**
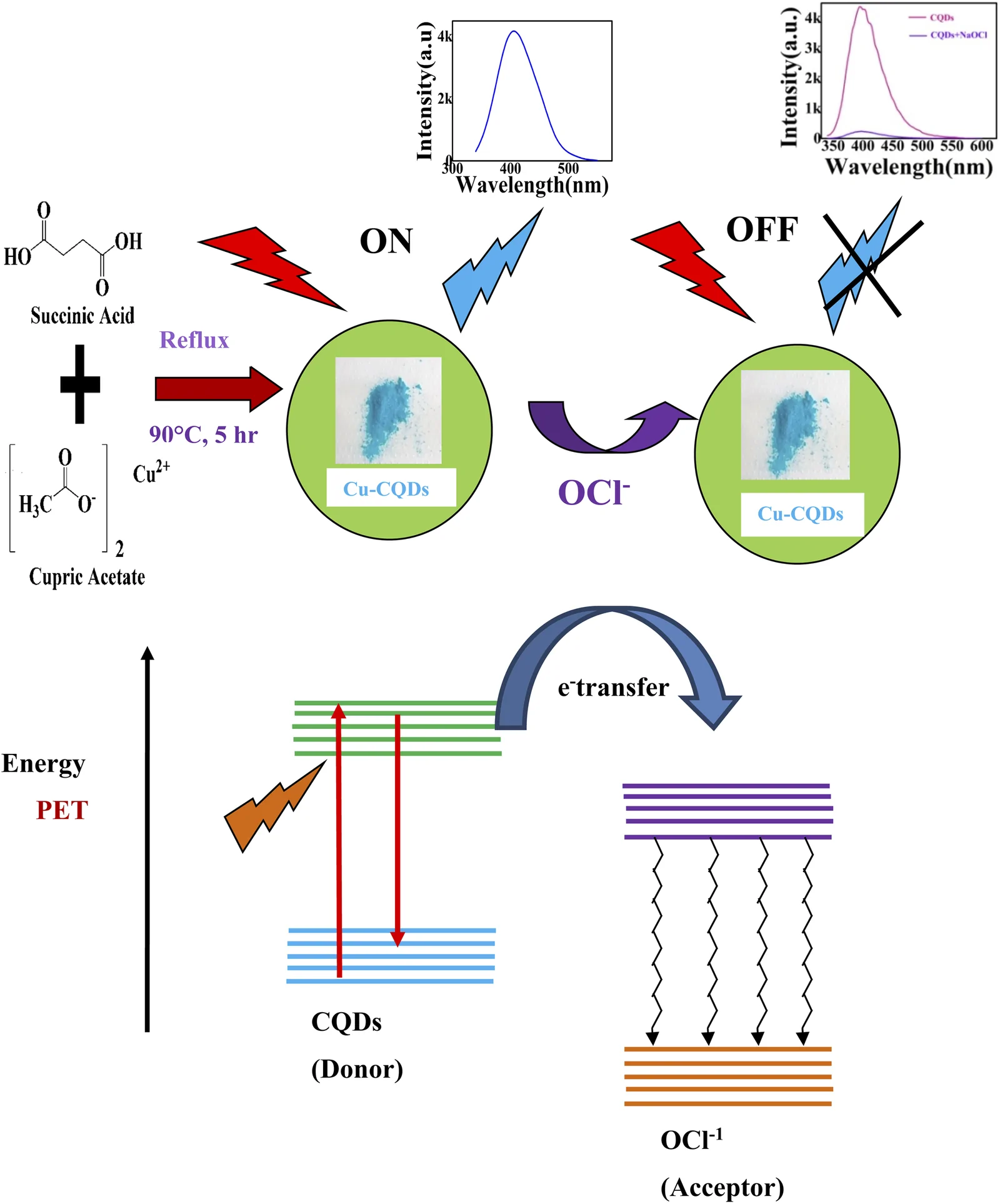
Schematic representation of sensing mechanism.

### 5.1. Selectivity of the detection system

The selectivity of the Cu-CQD probe toward OCl^−^ was evaluated by examining the fluorescence response in the presence of a wide range of potentially interfering metal ions, anions and other species under identical experimental conditions. The Cu-CQD concentration was maintained at 0.30 mg mL^−1^ at pH 8, and the fluorescence response was recorded after addition of the respective analytes. As shown in Fig. 10(a) and (b), most of the tested species produced negligible or comparatively small changes in the fluorescence intensity, whereas OCl^−^ caused pronounced fluorescence quenching.

**Fig. 10:**
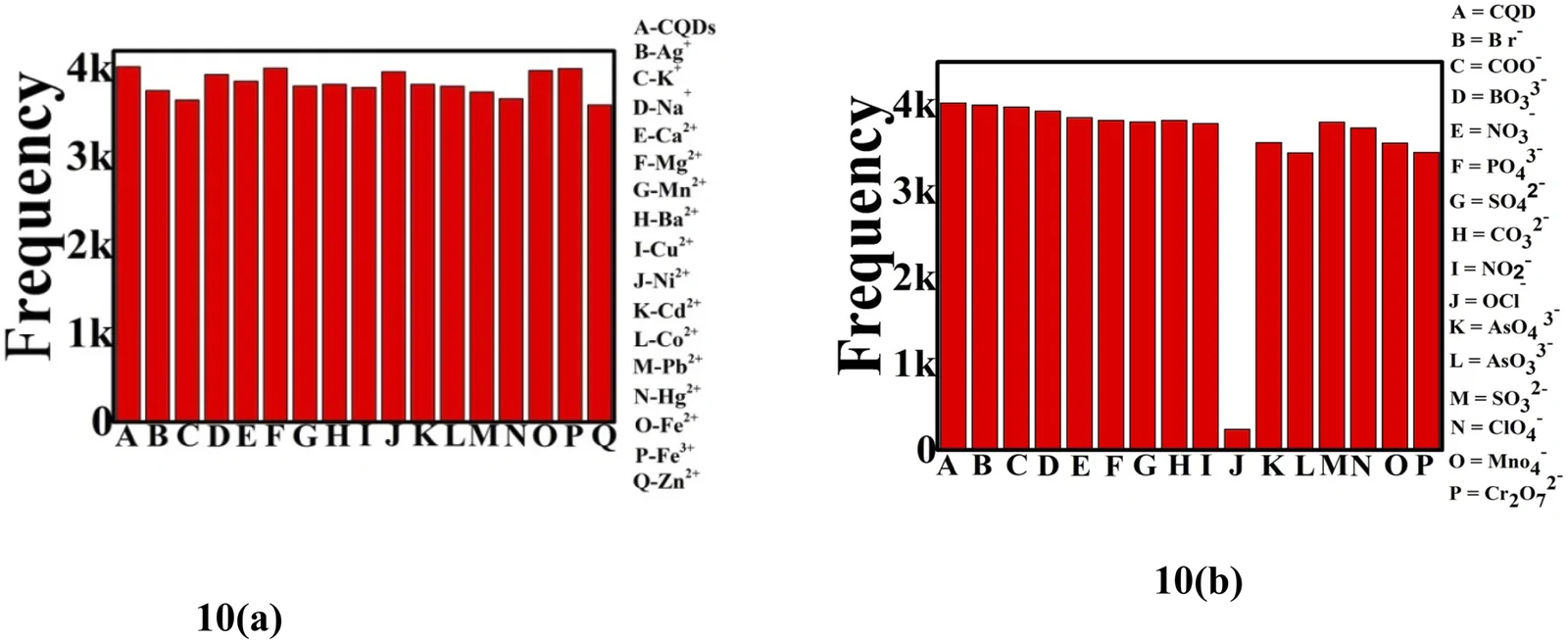
(a) and (b) effect of metal ions on the fluorescence property of Cu-CQDs.

The preferential response toward OCl^−^ can be attributed to the combination of its strong oxidizing ability and the chemically accessible surface states of the Cu-CQDs. The Cu-CQD surface contains oxygen-containing functionalities together with Cu-associated electronic states that can participate in excited-state interactions. Because OCl^−^ is a strong oxidizing species, its interaction with these emissive surface states can facilitate electron-transfer-assisted non-radiative deactivation of the excited Cu-CQDs, resulting in substantial fluorescence quenching. In contrast, most of the common inorganic ions and non-oxidizing species examined under the same conditions do not efficiently perturb these emissive states and therefore produce much smaller fluorescence changes.

### 5.2. TCSPC data analysis

In Fig. 11, we have shown the TCSPC decay transients of Cu-CQDs and Cu-CQDs after adding hypochlorite (OCl^−^) ions. As we wanted to see the comparative lifetime of our systems of interest so we excited both samples at the same wavelength. Excitation was done using 373 nm pulse diode and emission transients were recorded at 455 nm at a 54.7-degree magic angle. For the Cu-CQDs transients, the best-fit line was obtained assuming a bi-exponential decay curve. CQDs are intrinsically heterogeneous nanomaterials containing different local emissive environments associated with the carbonaceous core, oxygen-containing surface functionalities, defect states and Cu-associated electronic states. Photoexcited carriers located in these different environments may undergo radiative and non-radiative relaxation at different rates, giving rise to more than one fluorescence lifetime component. Thus, from the fitting of the curve, we obtained two lifetime components: one is relatively short (2.95 ns), and the other is long (7.16 ns). The short component has more weight (0.53) than the long component (0.47). The weighted average lifetime for Cu-CQDs was 4.93 ns. After adding hypochlorite the overall lifetime of the system decreased as evident from the emission transient of the system. The decay transient was best fitted assuming bi-exponential decay. Here we also obtained two lifetimes, one relatively short and another long. Short lifetime decreased from 2.95 ns to 1.70 ns. Long component also decreased from 7.16 ns to 5.90 ns. The addition of hypochlorite ions decreased the average lifetime of the excited state from 4.93 ns to 3.50 ns. The decrease in excited-state lifetime indicates that the nature of quenching is dynamic. This phenomenon may occur when the surface functional groups of Cu-CQDs promote this interaction. Basically, as the hypochlorite ion is a very strong oxidizing agent, it accepts electrons from Cu-CQDs in the excited state in a non-radiative pathway thus decreasing the excited state lifetime of the system. Such processes are known as photoinduced electron transfer (PET). The understanding of the electronic process involved in sensing is enhanced by band gap calculation (Fig. 7) alongside steady-state and time-resolved fluorescence findings. The significant quenching of fluorescence and reduction in excited-state life time when OCl^−^ is present suggest that the photoexcited state follows an extra-radiative deactivation route, aligning with the proposed PET mechanism (Table 3).

**Fig. 11:**
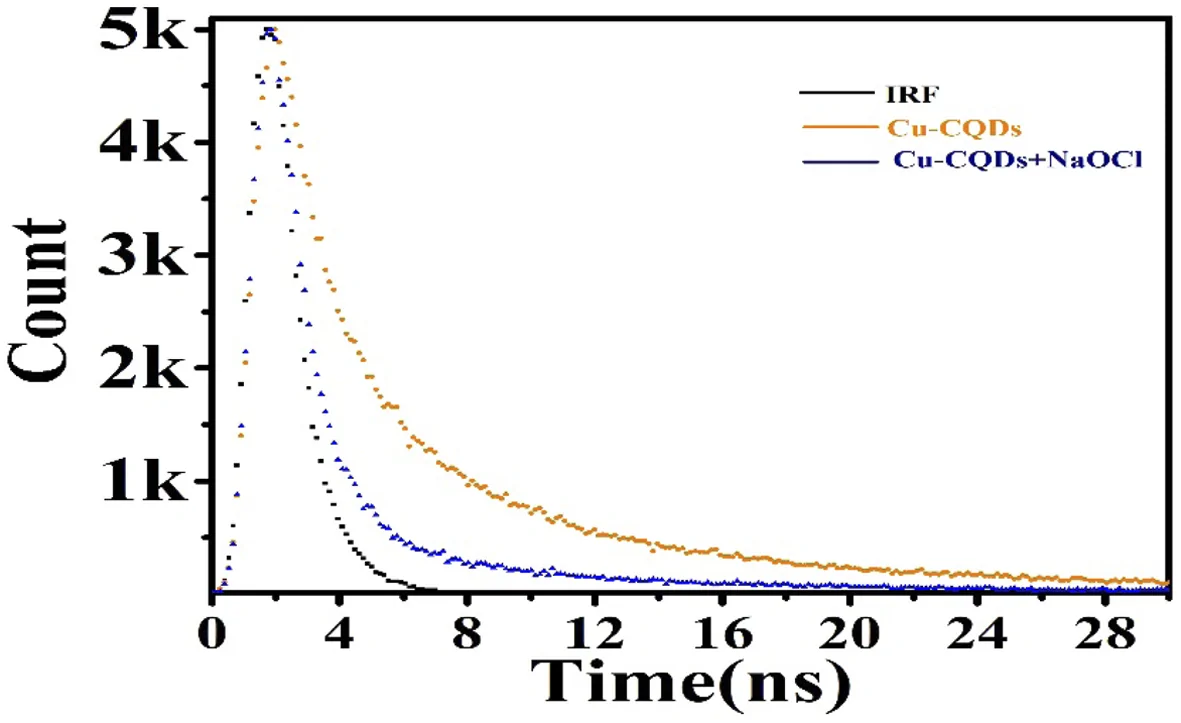
Picosecond transients for Cu-CQDs and Cu-CQDs + NaOCl.

**Table 3:** Lifetime data

Sample	*λ*_em_/nm	*τ*_1_/ns	*τ*_2_/ns	Average *τ*/ns	*a*_1_	*a*_2_
Cu-CQDs	373	2.95	7.16	4.93	0.53	0.47
Cu-CQDs + OCl^−^	373	1.7	5.9	3.5	0.57	0.43

### 5.3. Sensitivity of the detection system and limit of detection

The sensitivity of Cu-CQDs for OCl^−^ was recorded when different concentrations of OCl^−^ were added into the Cu-CQDs solution under optimal conditions. The fluorescence intensity of Cu-CQDs decreased gradually with increasing the concentration of OCl^−^ from 20–9000 nM. But the fluorescence peak did not shift (Fig. 12).

**Fig. 12:**
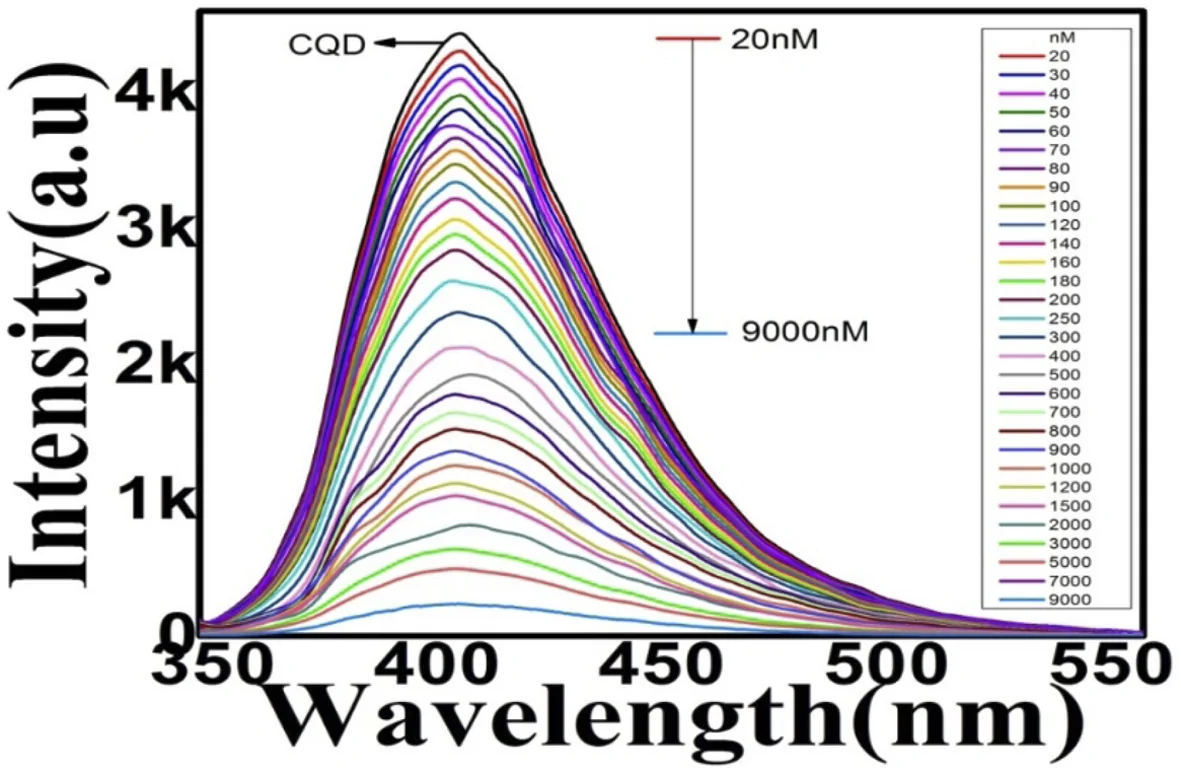
Finding detection limit for hypochlorite sensing.

The Stern–Volmer plot (Fig. 13) shows a linear relationship up to 400 nM with a correlation coefficient of 0.994. Using the correlation between hypochlorite ion and fluorescence intensity obtained from the Stern–Volmer plot at the low concentration range, the detection limit of OCl^−^ (using 3*σ*) is found to be ∼5.76 nM. The obtained detection limit value is much lower than the minimum (19.4 µM) and maximum (58.3 µM) levels of chlorine concentration permitted by WHO. The analytical performance of the present Cu-CQD probe is competitive with representative reported methods, although some reported CQD-based probes exhibit lower detection limits. [SI, Table S1].

**Fig. 13:**
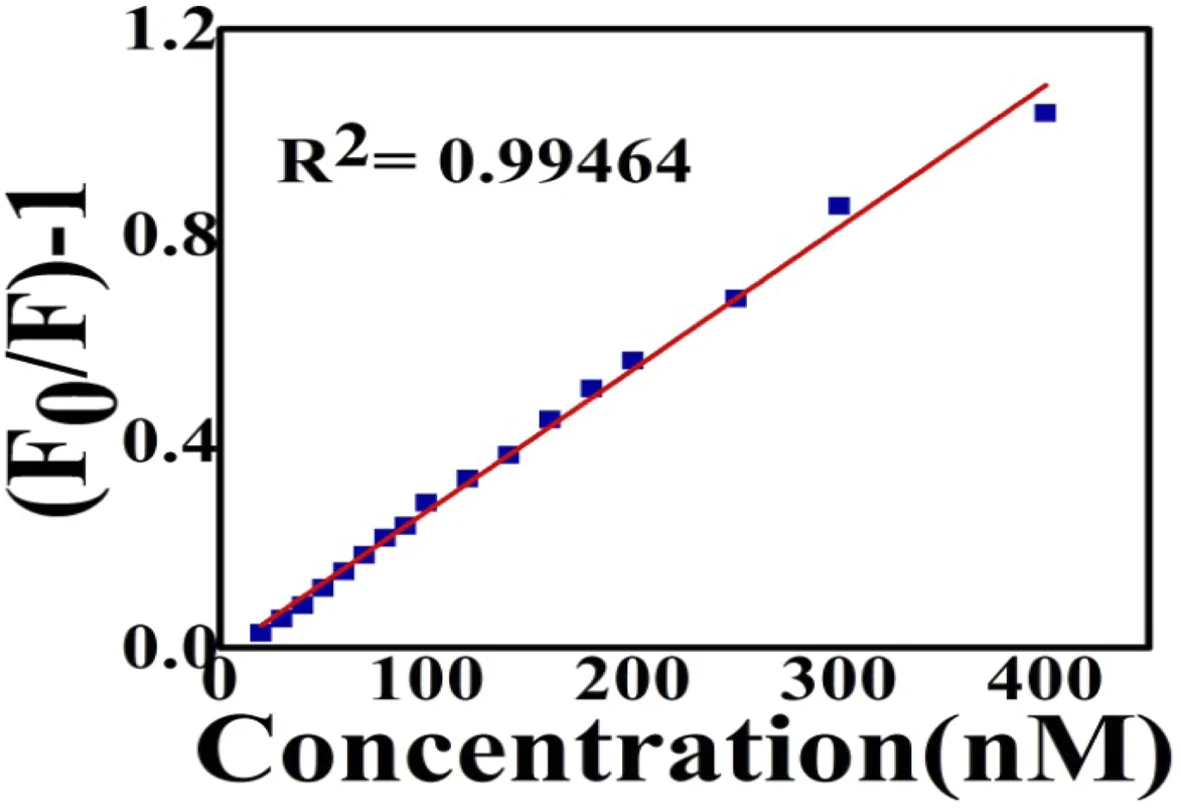
Stern–Volmer for linear detection range.

The analytical performance of the present Cu-CQD probe was compared with representative previously reported methods for hypochlorite detection, as summarized in Table S1. The reported methods employ different sensing platforms and detection principles, including colorimetric, chemiluminescence, electrochemical and fluorescence-based approaches. Heteroatom-doped CQDs have been reported to provide very low detection limits in both colorimetric and fluorescence sensing, whereas graphene quantum dots and modified carbon electrodes have also been employed for OCl^−^ determination over comparatively broad concentration ranges. Conventional CQD-based fluorescence probes provide the advantages of simple optical detection and aqueous compatibility, although their analytical sensitivity varies considerably depending on precursor composition, surface functionalization and sensing mechanism.

The present Cu-CQD probe exhibits a detection limit of 5.76 nM over a linear range of 0–400 nM. Although some previously reported heteroatom-doped or chemically engineered CQD probes exhibit lower detection limits, the present system provides a simple Cu-CQD platform with a sensitive fluorescence-off response toward OCl^−^ and is applicable to real-water analysis.

## 6. Application of Cu-CQDs for detecting OCl^−^ in real water samples

To evaluate the application of Cu-CQDs in some real samples, the sensor was applied to detect OCl^−^ in lake and tap water. We have collected lake water and tap water, and they were filtered through a 0.22 µm filter paper. Then it was further purified by centrifuging for 20 min at 3000 rpm. Then the water samples were mixed with Cu-CQDs (the concentration of Cu-CQDs is about 0.30 mg mL^−1^) and adjust pH 8 by using sodium phosphate buffer. After that, different concentrations of OCl^−^were added in this mixture and the change in fluorescence intensity of the Cu-CQDs was measured. The fluorescence intensity of Cu-CQDs at 340 nm decreased gradually with increasing the concentration of OCl^−^ (Fig. S5) up to 9000 nM.

As Table 4 shows, the obtained recovery values ranged from 92 to 103 percent so the method was successfully used for the determination of hypochlorite ion in real water samples.

**Table 4:** Hypochlorite ion sensing in real water samples with Cu-CQDs

Samples	Added (µM)	Found (µM)	Recovery (%)
Tap water	0	—	—
	5	4.6	92
	10	10.11	101
	15	15.48	103
Lake water	0	—	—
	5	5.07	101
	10	9.88	98
	15	15.09	100.6

## 7. Conclusion

We successfully synthesized copper-doped carbon quantum dots from succinic acid and cupric acetate using a simple and low-cost reflux method. The as-synthesized carbon quantum dots show good fluorescence property with 31.33% quantum yield. These Cu-CQDs can sense OCl^−^ ion selectively and sensitively by quenching the fluorescence emission. The quenching process is dynamic in nature as the fluorescence lifetime of Cu-CQDs decreases from 4.93 ns to 3.50 ns. The electrons of the excited state of the Cu-CQDs are transferred to the OCl^−^ ion *via* the PET mechanism. The limit of detection for OCl^−^ sensing is around 5.76 nM, which is significantly lower than the permissible value suggested by the World Health Organization.

## Author contributions

U. M. conceived the idea. A. D. and U. M. prepared Cu-CQDs. A. D. and U. M. performed the experiments, analyzed and tabulated the results. A. D. and U. M. wrote the first draft of the manuscript. A. D., S. I. revised the manuscript. All the authors contributed in writing the final draft of the manuscript.

## Conflicts of interest

There is no conflicts of interest among the authors to declare.

## Supplementary Material

RA-016-D6RA05419F-s001

## Data Availability

The authors confirm that the data supporting the findings of this study are available within the research article. Supplementary information (SI) is available. See DOI: https://doi.org/10.1039/d6ra05419f.
