# Supplementary material for: A highly sensitive carbon quantum dots sensor for hypochlorite ion detection through fluorescence-off method
Source: RSC Adv. 2026 Oct 14;16(47):62908–16. doi: 10.1039/d6ra05419f (PMC13664704; doi:10.1039/d6ra05419f)
Supplement: Supplementary file 1 [file RA-016-D6RA05419F-s001.pdf]

Supplementary Information (SI) for RSC Advances.
This journal is © The Royal Society of Chemistry

A Highly Sensitive Carbon Quantum Dots Sensor for Hypochlorite Ion Detection through Fluorescence-Off Method

Ankita Dutta[1], Sahidul Islam[2] Ujjwal Mandal[1*]

[1]Department of Chemistry, The University of Burdwan, Golapbag, Burdwan-713104

[2]Department of Chemistry, Murshidabad Maharaja Krishnanath University,Berhampore, 742101

E-mail: umandal@chem.buruniv.ac.in

Supplementary Informations (ESI):

4.3 EDX Analysis:

The composition and spatial arrangement of synthesized carbon quantum dots have been studied through EDX analysis and elemental mapping. Fig.S1 and Fig. S2(A-E) show EDX and elemental mapping, respectively. From Table S1, we can conclude that our synthesized CQDs contain Carbon, Oxygen, and Copper as element having weight percentage 49.98, 45.53, and 4.50 and atomic percentage 58.80, 40.20, and 1.00, respectively. The combined XRD, EDX and elemental-mapping results support the formation of the carbonaceous Cu-CQD material and confirm the presence of Cu in the synthesized nanoparticles. EDX analysis revealed C, O and Cu with weight percentages of 49.98%, 45.53% and 4.50%, respectively, while elemental mapping showed the spatial distribution of these elements within the analyzed particles. These results provide compositional evidence for the incorporation of Cu into the synthesized CQDs

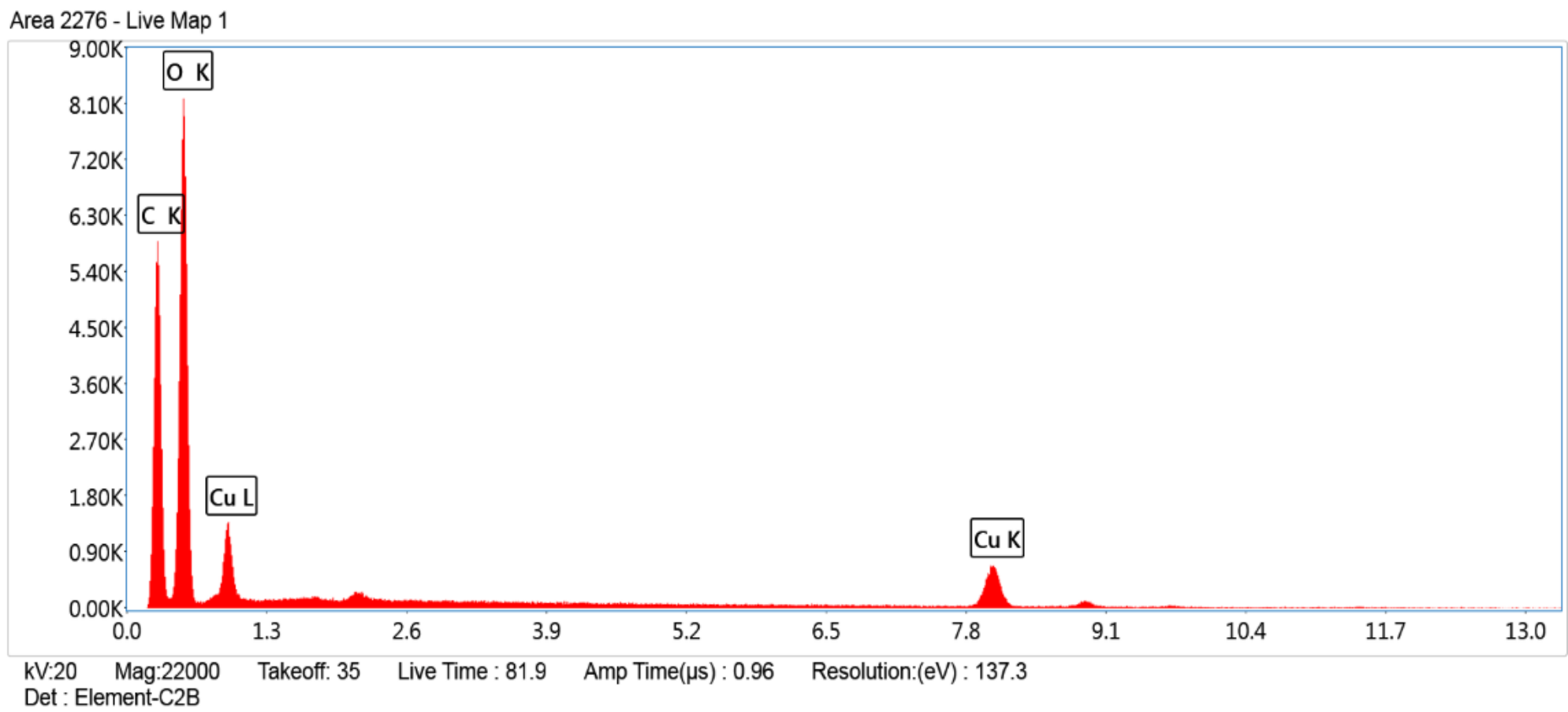

Fig. S1 EDX analysis of Cu-CQDs

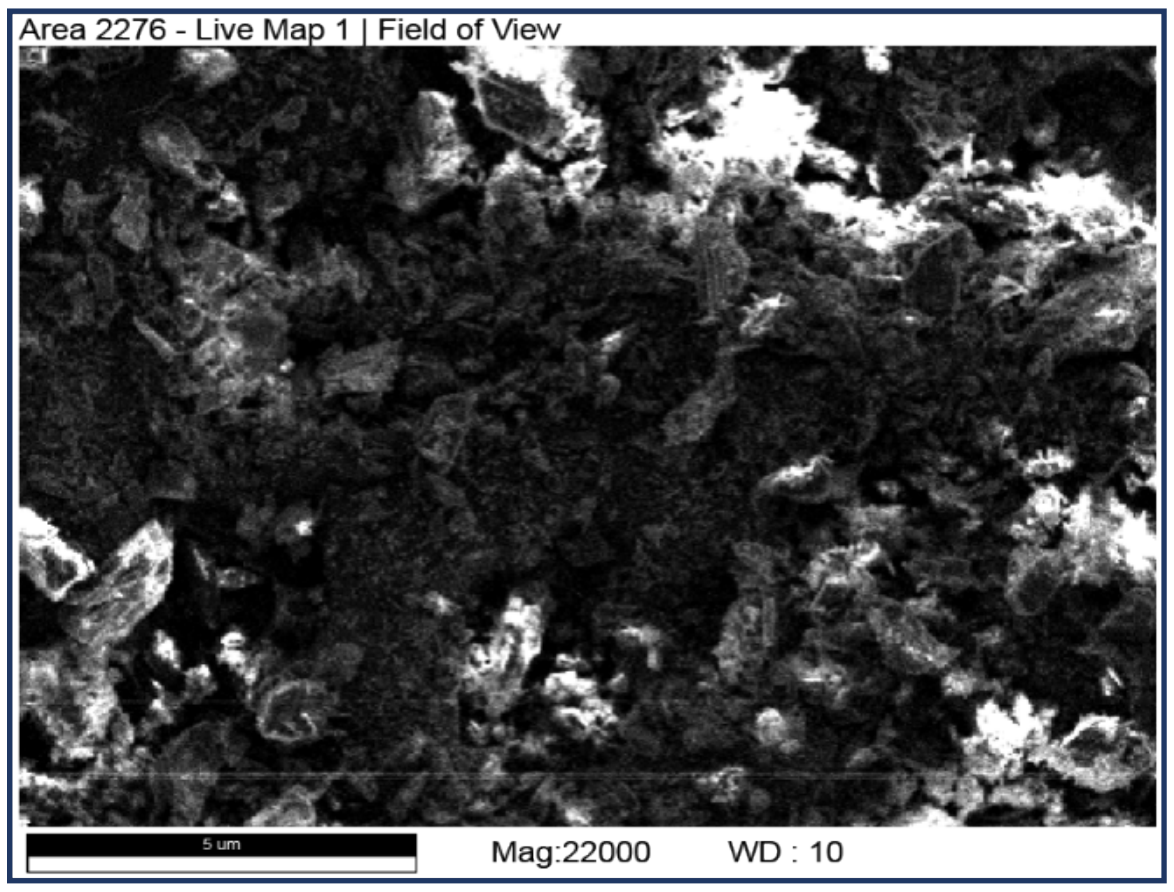

Fig. S2 (A)

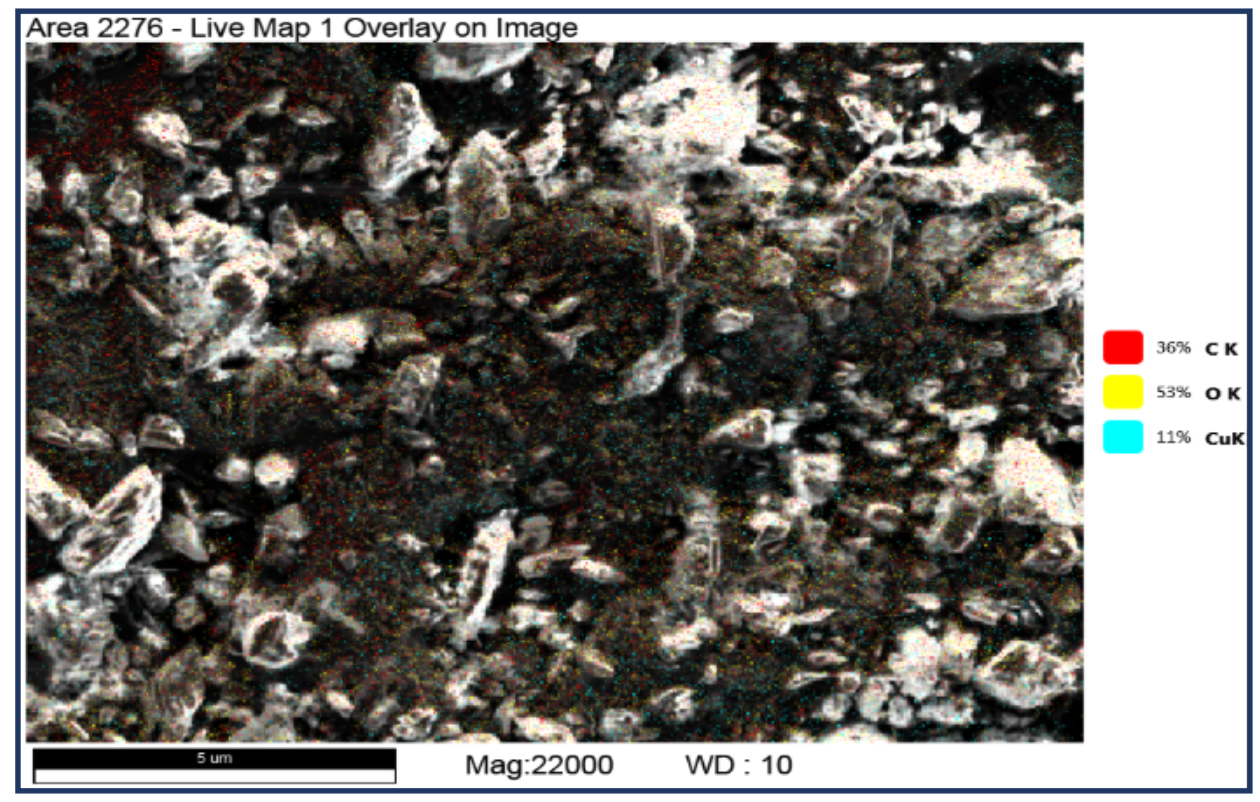

Fig. S2 (B)

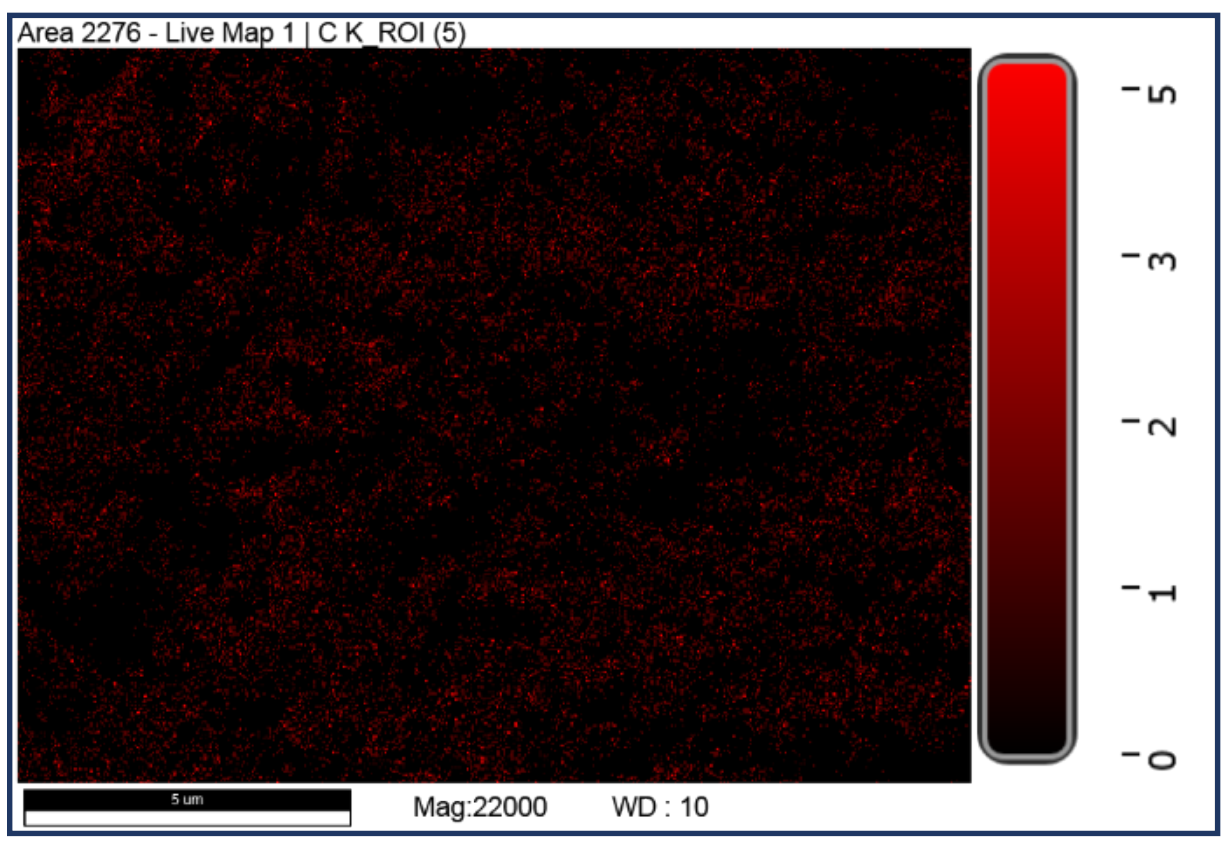

Fig. S2 (C)

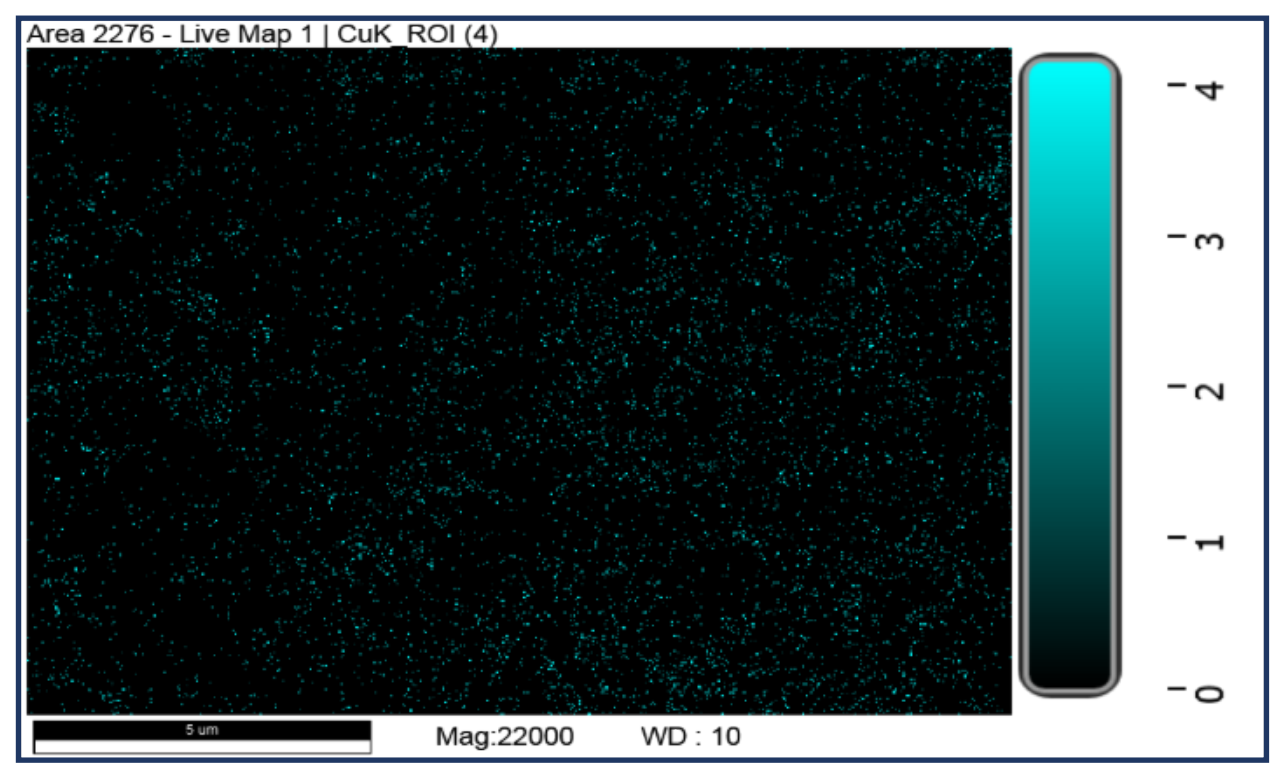

Fig. S2 (D)

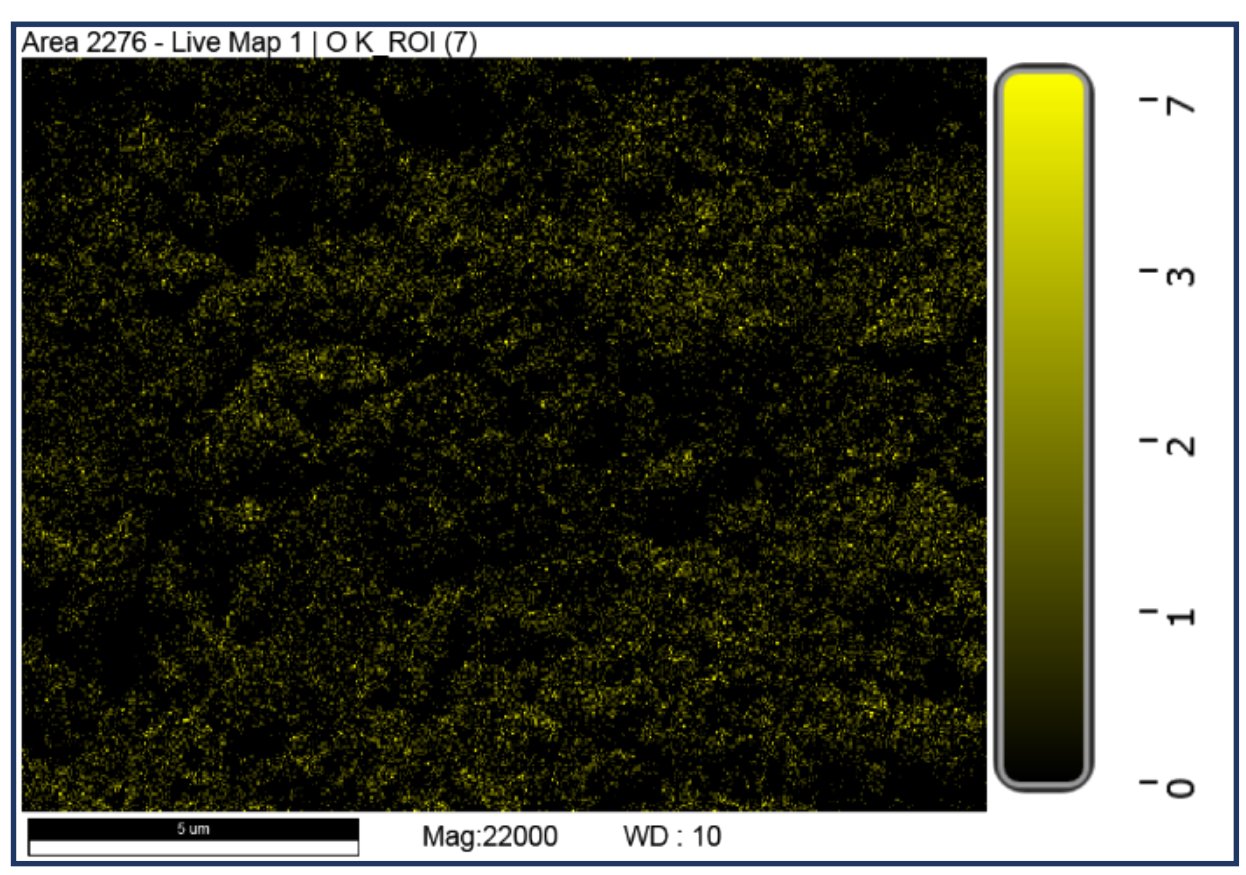

Fig. S2 (E)

Fig. S2 (A)-(E) Elemental Mapping of Cu-CQDs

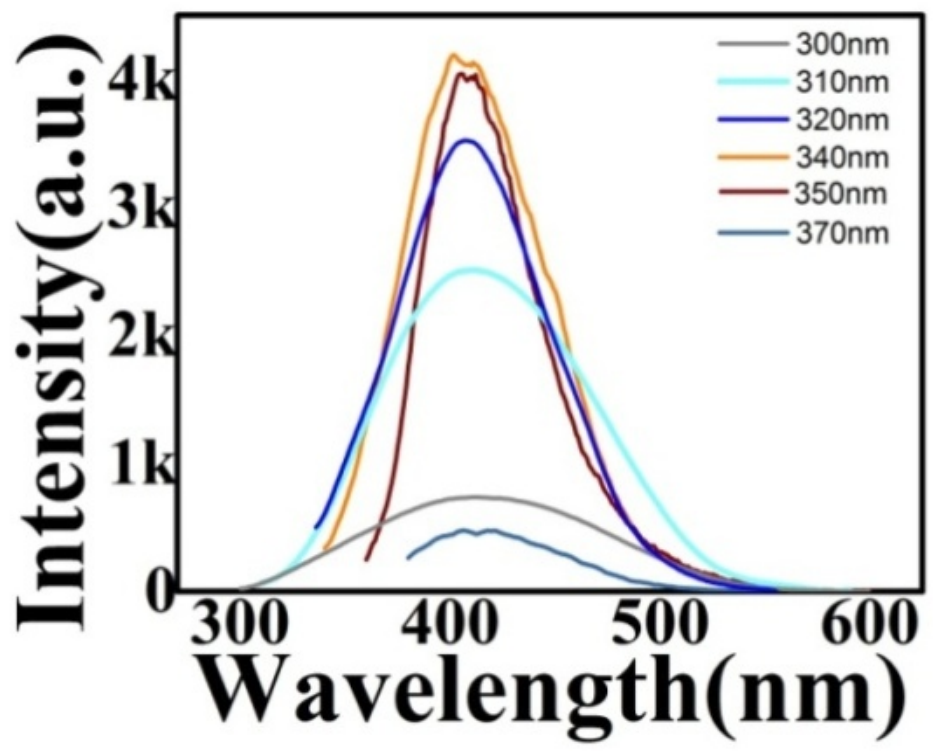

Fig. S3 Excitation wavelength dependence of Cu-CQDs

emission spectra

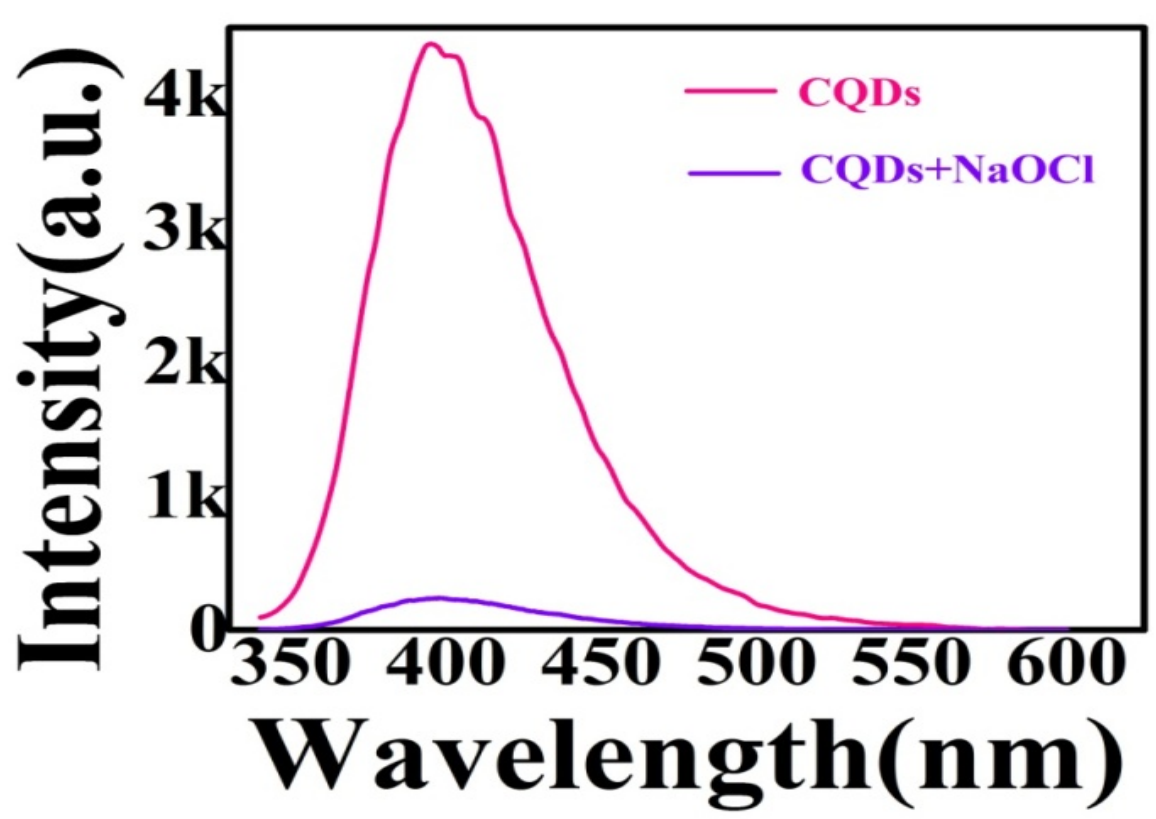

Fig. S4: Emission spectra of synthesized Cu-CQDs with

and without NaOCl

Table S1: Hypochlorite Detection Limit from different techniques/quantum dots:

Sl. No	Method	Materials	Linear range	Detection limit	References
1	Colorimetry	Si, N, P doped CQDs	0.1–1.3 μM.	1.44 nM	1 (ref 31 in main manuscript)
2	Fluorescence	Si, N, P doped CQDs	0.1–1.4 μM	0.79 nM	1
3	DPV	Si, N, P doped CQDs	0.1–0.9 μM	1.70 nM	1
4	Fluorescence	N, P-CQDs	0.1–0.9 μM	2.54 nM	1
5	Colorimetry	N, P-CQDs	0.1–0.9 μM	3.84 nM	1
6	DPV	N, P-CQDs	0.1–0.9 μM	4.10 nM	1
7	Fluorescence	Carbon Quantum Dots	1-1500 μM	36.54 nM	2
8	Electrochemistry	Polymelamine-modified carbon electrode	10 μM-7 mM	5.5 μM	3
9	Chemiluminescence	Graphene Quantum Dots	0.5 μM -1 mM	0.3 mM	4
10	Fluorescence	Glutathione functionalized molybdenum disulfide Quantum Dots	□1-20 μΜ	11.5 nM	5

11	Fluorescence	Carbon Quantum Dots	0-400 nM	5.76 nM	This work

Synthesis of CQDs.

1, 2, 3 In this study Si, N, P- doped CQDs were prepared using (3-aminopropyl) trimethoxy silane (APTMOS), urea, citric acid, and H_3PO_4 by one-pot hydrothermal method.

4, 5, 6 N, P-doped CQDs were made using citric acid, urea, H₃PO₄ and dimethyl formamide under an inert condition.

7. In this paper Graphene Quantum Dots were prepared from Glucose using pyrolysis method.

9. Here, The Carbon Quantum Dots were prepared from Glutathione through hydrothermal method at 200 °C for 12 h

10. In this work G-MoS2 (glutathione functionalized molybdenum disulfide quantum dots). Quantum Dots was prepared through hydrothermal method. 0.15 g Na2MoO4⋅2H2O and 15 mL Milli-Q water, was mixed together and pH 6.5 was adjusted using 0.1 M HCl, then 0.258 g glutathione and 30 mL Milli-Q water was added. and this total mixture was shifted to Teflon-lined autoclave for 12 h at 200 °C

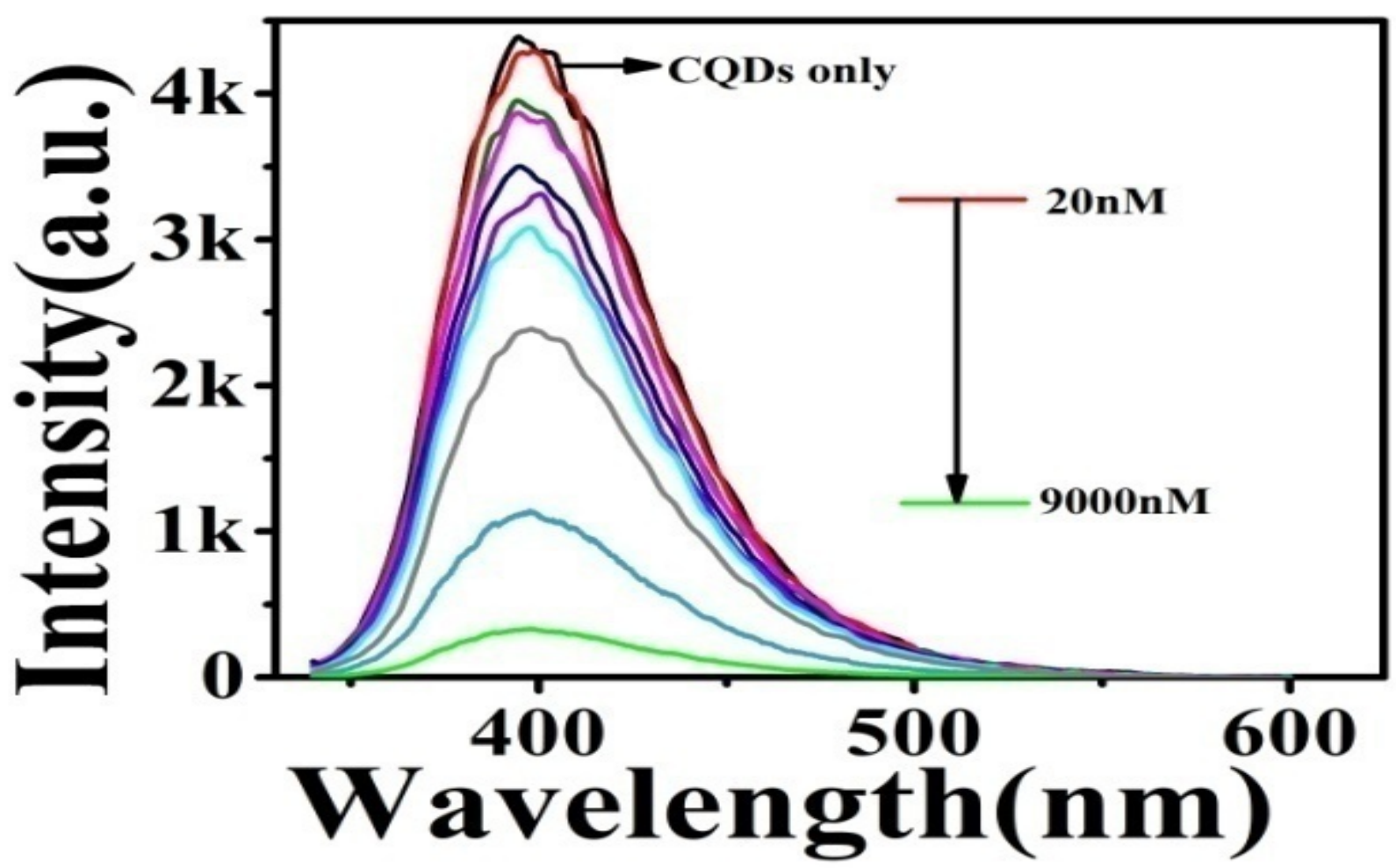

Fig. S5 Finding the detection limit for hypochlorite sensing in real water sample

1. Hafez NS, Hegazy MBZ, Khalifa A, et al (2026) Multi-heteroatom doped carbon quantum dots for ultrasensitive detection of hypochlorite ions in aqueous media. Microchem J 224:117631. https://doi.org/10.1016/j.microc.2026.117631
2. Shao K, Guo L, Zhong Y, et al (2024) Carbon Quantum Dots for Rapid and Ratiometric Fluorescence Determination of Hypochlorite. ACS Appl Nano Mater 7:8645–8654. https://doi.org/10.1021/acsanm.3c06131
3. Senthilkumar K, Zen J-M (2014) Free chlorine detection based on EC' mechanism at an electroactive polymelamine-modified electrode. Electrochem commun 46:87–90. https://doi.org/10.1016/j.elecom.2014.06.018
4. Hallaj T, Amjadi M, Manzoori JL, Shokri R (2015) Chemiluminescence reaction of glucose-

derived graphene quantum dots with hypochlorite, and its application to the determination of free chlorine. Microchim Acta 182:789–796. https://doi.org/10.1007/s00604-014-1389-0

5. Zhong Y, Guo L, Zou Y, et al (2023) Rapid and ratiometric fluorescent detection of hypochlorite by glutathione functionalized molybdenum disulfide quantum dots. Spectrochim Acta Part A Mol Biomol Spectrosc 295:122649. https://doi.org/10.1016/j.saa.2023.122649
